# Endurance Training Provokes Arrhythmogenic Right Ventricular Cardiomyopathy Phenotype in Heterozygous Desmoglein-2 Mutants: Alleviation by Preload Reduction

**DOI:** 10.3390/biomedicines12050985

**Published:** 2024-04-30

**Authors:** Larissa Fabritz, Lisa Fortmueller, Katja Gehmlich, Sebastian Kant, Marcel Kemper, Dana Kucerova, Fahima Syeda, Cornelius Faber, Rudolf E. Leube, Paulus Kirchhof, Claudia A. Krusche

**Affiliations:** 1 University Center of Cardiovascular Science and Department of Cardiology, University Heart and Vascular Center, University Hospital Hamburg Eppendorf, 20246 Hamburg, Germany; lisa.fortmueller@googlemail.com (L.F.); p.kirchhof@uke.de (P.K.); 2 German Center for Cardiovascular Research (DZHK), Partner Site Hamburg/Kiel/Lübeck, 20246 Hamburg, Germany; 3 Institute of Cardiovascular Sciences, University of Birmingham, Birmingham B15 2TT, UK; k.gehmlich@bham.ac.uk (K.G.); marcel.kemper@ukmuenster.de (M.K.); fahima.syeda@gmail.com (F.S.); 4 Department of Cardiology, Section of Rhythmology, University Hospital Muenster, 48149 Münster, Germany; myocyt@gmail.com; 5 Division of Cardiovascular Medicine, Radcliffe Department of Medicine, University of Oxford, Oxford OX1 2JD, UK; 6 Institute for Molecular and Cellular Anatomy (MOCA), RWTH Aachen University, 52074 Aachen, Germany; skant@ukaachen.de (S.K.); rleube@ukaachen.de (R.E.L.); 7 Clinic of Radiology, Translational Research Imaging Center (TRIC), University of Muenster, 48149 Münster, Germany; faberc@uni-muenster.de

**Keywords:** desmoglein 2, preload-reducing therapy, arrhythmogenic right ventricular cardiomyopathy (ARVC), mouse model, arrhythmogenic cardiomyopathy, endurance exercise, intercalated disk, desmosome

## Abstract

Desmoglein-2 mutations are detected in 5–10% of patients with arrhythmogenic right ventricular cardiomyopathy (ARVC). Endurance training accelerates the development of the ARVC phenotype, leading to earlier arrhythmic events. Homozygous *Dsg2* mutant mice develop a severe ARVC-like phenotype. The phenotype of heterozygous mutant (*Dsg2^mt/wt^*) or haploinsufficient (*Dsg2^0/wt^*) mice is still not well understood. To assess the effects of age and endurance swim training, we studied cardiac morphology and function in sedentary one-year-old *Dsg2^mt/wt^* and *Dsg2^0/wt^* mice and in young *Dsg2^mt/wt^* mice exposed to endurance swim training. Cardiac structure was only occasionally affected in aged *Dsg2^0/wt^* and *Dsg2^mt/wt^* mice manifesting as small fibrotic foci and displacement of Connexin 43. Endurance swim training increased the right ventricular (RV) diameter and decreased RV function in *Dsg2^mt/wt^* mice but not in wild types. *Dsg2^mt/wt^* hearts showed increased ventricular activation times and pacing-induced ventricular arrhythmia without obvious fibrosis or inflammation. Preload-reducing therapy during training prevented RV enlargement and alleviated the electrophysiological phenotype. Taken together, endurance swim training induced features of ARVC in young adult *Dsg2^mt/wt^* mice. Prolonged ventricular activation times in the hearts of trained *Dsg2^mt/wt^* mice are therefore a potential mechanism for increased arrhythmia risk. Preload-reducing therapy prevented training-induced ARVC phenotype pointing to beneficial treatment options in human patients.

## 1. Introduction

Arrhythmogenic right ventricular cardiomyopathy (ARVC [1]) is an inherited cardiomyopathy that manifests with right ventricular arrhythmias and increased risk of sudden cardiac death and heart failure [2,3,4,5]. In humans the most common mode of inheritance is autosomal-dominant, although recessive hereditary traits are also found [6,7,8].

Major arrhythmic events can occur early in the course of the disease, the so-called concealed phase [9]. The prevalence of ARVC is estimated at 1:5000–1:1000. Notably, ARVC is a common cause of sudden death in young people, with some regional variability [10,11,12,13]. Disease diagnosis or manifestation is most common in the second to fifth decade of life, but children can also be affected by the disease [14,15,16].

About 50% of ARVC patients carry a mutation in genes encoding one of the five essential cardiac desmosomal proteins: the desmosomal cadherins desmoglein (DSG)-2 and desmocollin-2 and the armadillo repeat-containing proteins plakoglobin and plakophilin (PKP)-2, as well as the plaque protein desmoplakin ([6,8,17], graphical abstract). Desmosomes are a principal component of the intercalated disc, and proper desmosome function is crucial for the mechanical and electrical coupling of cardiomyocytes [18]. By anchoring the desmin filament network at the intercalated discs, desmosomes are involved in the maintenance of cardiomyocyte structures [19] and are increasingly recognized as important signaling hubs [20,21]. Intercalated discs and their components attain full functional structure and maturity during postnatal life [22,23]; however, aging negatively affects their structure and function [24].

ARVC progressively affects both the right and left ventricles, with the right ventricle (RV) often being affected first [4,13,25,26,27]. Overall, the right ventricle is more susceptible to wall stress than the left ventricle (LV). Right ventricular volume overload and high-end systolic right ventricular wall stress (ES-sigma) during endurance training are suspected to trigger arrhythmia or sudden cardiac death or accelerate the onset and progression of ARVC in mutation carriers [28,29,30,31,32]. This was indirectly demonstrated when the introduction of a screening program for professional athletes in Italy reduced the incidence of sudden cardiac death [26]. The clinical observation of accelerated disease progression by exercise was first experimentally verified by our group in a murine model with reduced plakoglobin levels, which was subjected to an incremental seven-week-long endurance swim training [33]. Based on these experimental findings, ARVC patients or mutation carriers are advised to avoid physical activity with moderate- to high-intensity components, long-duration exercise, and/or any activity that provokes palpitations or syncope in an individuum [30,34]. However, discussions continue about which level of exercise might still be safe and if there are differences between the pathogenic gene variants [35,36,37].

Of ARVC patients with desmosomal mutations, 5–10% carry a mutation in one *DSG2* allele [38,39]. Mutational hot spots are mostly located in exons encoding the extracellular domains that facilitate cis- and trans-interactions [17,40]. In addition to point mutations that induce amino acid exchanges, there are mutations that generate stop codons, resulting in truncated proteins and null mutants [17,41]. The mutation type and localization can potentially reduce protein levels and/or corrupt DSG2 protein functions, like adhesion strength, binding kinetics, or intercalated disc-driven signaling pathways due to altered binding sites for interacting partner proteins [42,43,44,45].

**Figure 1:**
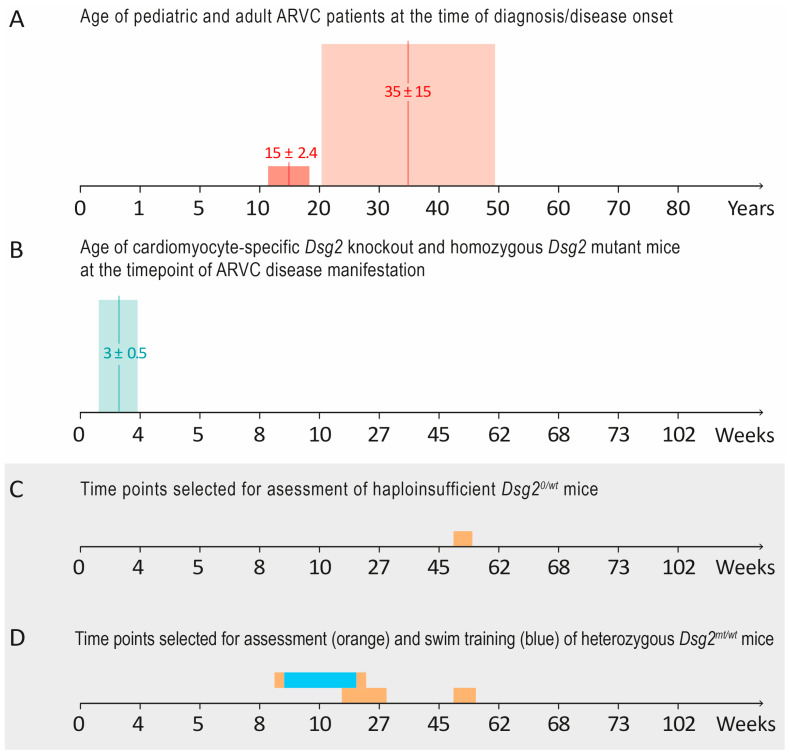
Summarizes the rationale for the models and time points used in this study. (**A**) The time points of ARVC manifestation in human patients (age ± SD) [14]. (**B**) The corresponding mouse ages (in weeks) according to the study by Wang and coworkers [60] and the time points of structural ARVC onset with cardiomyocyte death, inflammation, and fibrotic remodeling that we observed in homozygous *Dsg2* mutant [48] and cardiomyocyte-specific *Dsg2* knockout mice [42]. These mouse models mimicked ARVC patients with disease onset in early childhood but not in patients with disease onset during their second to fifth decade of life. (**C**,**D**) We therefore investigated haploinsufficient *Dsg2^0/wt^* mice [59] and heterozygous mutant *Dsg2^mt/wt^* mice [48] at the indicated ages (bars in orange) to assess whether they were useful models for the concealed phase and/or an ARVC onset in adulthood. We first analyzed the histomorphology and cardiac function of sedentary one-year-old mice, i.e., an age corresponding to 40- to 50-year-old humans. We then assessed sedentary *Dsg2^mt/wt^* mice at 16–29 weeks, which corresponded to 25- to 30-year-old humans. Since no obvious ARVC phenotype was detected in this age range, a seven-week-long endurance swim training, which is an aerobic exercise known to induce physiological cardiac growth (blue bar), was initiated at the age of 8–12 weeks to provoke an ARVC phenotype.

To date, several mouse models based on homozygous mutation of the endogenous *Dsg2* allele have been generated and assessed [46,47]. Homozygous *Dsg2* mutant or conditional *Dsg2* knockout mice recapitulate major features of biventricular ARVC with cardiomyocyte death, aseptic inflammation, cardiomyocyte hypertrophy, autophagy, fibrosis [42,47,48,49,50,51,52,53,54,55], and numerous changes in related signaling pathways [44,53,54,55,56]. As expected, endurance swim training and psychosocial stress aggravated ARVC disease progression in a homozygous mutant *Dsg2* mouse model [55,57]. Since homozygous *Dsg2* mutation triggers a severe structural ARVC phenotype in mice aged 2 to 3 weeks, they are suitable models for studying the rare condition of ARVC onset in childhood [58]. However, they do not fully recapitulate the disease course of most heterozygous childhood and adult patients with ARVC (Figure 1A,B). To better mimic the constellation encountered in the majority of human ARVC patients, we therefore used two heterozygous mouse models in this study. They were derived from alleles that were generated in our lab [48,59]. The haploinsufficient *Dsg2^0/wt^* mouse contained a mutant *Dsg2* allele with a deletion of exons 7 and 8 resulting in a small aminoterminal DSG2 fragment encompassing only extracellular domain EC1 and a part of EC2 (see [59]). Since the encoded residual protein was not integrated into the membrane, we referred to it as loss-of-function allele “0”. The mutant *Dsg2^mt/wt^* mouse, on the other hand, produced a mutant DSG2, which lacked parts of EC1 and EC2 but was still integrated in the membrane and delivered to the intercalated disc [48]. We referred to this allele as “mt”. We first investigated whether sedentary haploinsufficient and heterozygous *Dsg2* mutant mice developed an ARVC phenotype at the age of one year that resembles a 40- to 50-year-old human ([58,60]; Figure 1C,D; graphical abstract). We detected only subtle histomorphological changes. We then explored whether swim training led to the manifestation of an ARVC-like phenotype already in young adult mice (Figure 1D). Since this was the case, we wanted to find out whether preload-reducing therapy prevented this training-induced phenotype, as we have shown before in another mouse model ([33,61], graphical abstract).

## 2. Materials and Methods

### 2.1. Experimental Animals and Training Protocol

The generation of haploinsufficient *Dsg2^0/wt^* mice and *Dsg2^mt/mt^* mice lacking exons 4–6 has been described [48,59]. All mice were bred in the Institute for Laboratory Animal Science & Experimental Surgery, Medical Faculty, RWTH-Aachen University. Animals were housed under standardized conditions and had free access to water and a standard rodent lab diet (Ssniff). The experiments were conducted in accordance with the guidelines for the care and use of laboratory animals and approved by the Ministry for Climate Protection, Environment, Agriculture, Conservation and Consumer Protection of the State of North Rhine-Westphalia (LANUV, reference number 8.87-50.10.37.09.114 and A4 notifications for killing animals for scientific purposes).

First, we compared the heart morphology and cardiac function of sedentary 16- to 26-week-old and one-year-old *Dsg2^mt/wt^* mice and sedentary one-year-old haploinsufficient *Dsg2^0/wt^* mice with their respective wild-type controls. The one-year time point was chosen since an ARVC phenotype was previously observed by us in another heterozygous ARVC mouse model [33]. Young adult *Dsg2^mt/wt^* and *Dsg2^WT^* control mice with two wild-type *Dsg2* alleles were subjected to endurance swim training and multiple analyses of heart function. For these experiments, the mice were transferred to the animal facility of the University Hospital Münster.

### 2.2. Echocardiography

Echocardiography was performed during anesthesia with 1.5–2% isoflurane + oxygen in one-year-old *Dsg2^0/wt^* mice and their *Dsg2^WT^* littermates (*n* = 6 for each genotype). In *Dsg2^mt/wt^* and *Dsg2^WT^* littermates, echocardiography was performed at the ages of 16 and 26 weeks (*n* = 11 per genotype), which corresponded to 20–30 years in humans, to screen for a potential impairment of cardiac function. *Dsg2^mt/wt^* and *Dsg2^WT^* littermates taking part in the endurance swim training program underwent echocardiography before and after training (*Dsg2^mt/wt^*: *n* = 25, including 11 preload reduction treated mice; *Dsg2^WT^*: *n* = 24, including 10 preload reduction treated mice) to study the effects of training with and without therapy. Left and right ventricular dimensions and functions were measured using a dedicated small animal ultrasound unit (Vevo 2100, FujiFilmVisualSonics, Toronto, ON, Canada) and analyzed following validated protocols for left and right ventricular size and function [33].

### 2.3. Endurance Swim Training and Preload-Reducing Therapy

Endurance swim training is a forced training modality known to induce physiological cardiac growth [62]. Here we assessed whether this type of training triggered an ARVC phenotype in *Dsg2^mt/wt^* mice. *Dsg2^mt/wt^* and *Dsg2^WT^* littermate controls were recruited for incremental endurance swim training at ages of 8 to 12 weeks. Male and female mice were used in these experiments.

Group swimming training sessions were run on six days a week, starting with a five-minute swim interval that gradually increased to 90 min per day for seven weeks (Supplemental Table S1 [33]). To prevent hypothermia, the water temperature was kept at 35 °C. Animals swam spontaneously but were allowed to temporarily rest. Mice were under permanent observation. Echocardiography was performed directly before and after completion of the training period. To verify that the endurance swim training was effective, the left ventricular weight/body weight index was calculated by measured parameters during echocardiography. ECGs were taken after the training period.

Furthermore, we studied whether preload reduction by administration of nitrates and diuretics [61] was suitable to prevent right ventricular dilation induced during endurance swim training in *Dsg2^mt/wt^* mice (graphical abstract; *Dsg2^mt/wt^*: *n* = 25, including 11 treated mice; *Dsg2^WT^*: *n* = 24, including 10 treated mice). Load-reducing therapy consisted of the loop diuretic furosemide (4 mg/kg/day) and isosorbide dinitrate (20 mg/kg for 15 h/day) alternating with molsidomine (8 mg/kg for 9 h/day) to avoid nitrate tolerance. All drugs were administered via drinking water. Fluid intake was monitored daily to adapt drug concentrations. Placebo therapy consisted of pure drinking water.

### 2.4. Electrocardiography

Surface electrocardiography (ECG) was recorded non-invasively (emka TECHNOLOGIES—Paris, France) in mice anaesthetized with urethane (2 mg/kg) before heart extraction. ECGs were recorded for 2 min and scanned for arrhythmias. Averaged signals up to 100 single beats at similar heart rates were analyzed for common values. ECGs were performed in one-year-old *Dsg2^0/wt^* (*n* = 5) and *Dsg2^WT^* littermates (*n* = 4) to assess the effects of genotype and aging on cardiac electrophysiology. In heterozygous *Dsg2^mt/wt^* mutant mice, ECGs were performed on 29-week-old resting animals (*n* = 8 for *Dsg2^mt/wt^*; *n* = 7 for *Dsg2^WT^* littermate controls) to gain reference values for the training and therapy group. In the mice subjected to swim training, ECGs were studied after training (*Dsg2^mt/wt^*: *n* = 23, including 10 treated mice; *Dsg2^WT^*: *n* = 17, including 5 treated mice). In these mice, late ventricular activation in the ECG was measured from the S wave trough (S_min_) to the next peak (J_peak_). More detailed information on activation was gathered by measuring ventricular activation times analyzed in the intact heart.

### 2.5. Assessment of Cardiac Electrophysiology in Isolated Langendorff Hearts

Hearts were excised under terminal anesthesia using urethane (2 mg/kg) and mounted on a Langendorff apparatus (Hugo Sachs Elektronik Harvard Apparatus, March-Hugstetten, Germany). Right atrial and right ventricular pacing was performed via an octopolar catheter (NuMED, Hopkinton, MA, USA). After fixfrequent S1 pacing, a single encroaching S2 extrastimulus was applied to test whether ventricular arrhythmia was inducible using the double pacing threshold. Ventricular activation times were analyzed (emka TECHNOLOGIES, Paris, France). After the electrophysiological tests, the hearts were dissected for immunoblot analysis or histological assessments [33,61].

### 2.6. Gross Morphological and Histological Examination

Cardiac morphology was studied in 48- to 73-week-old (median 52 weeks) mice, which we refer to as “one year old” for simplicity’s sake. The analyses were performed in heterozygous *Dsg2^mt/wt^* mice (*n* = 79) and age-matched wild-type controls (*n* = 17) and haploinsufficient *Dsg2^0/wt^* mice (*n* = 62) and their wild-type *Dsg2^WT^* littermates (*n* = 36).

Histological assessments of hearts after training and Langendorff analysis were performed in seven *Dsg2^WT^* mice (*n* = 4 with therapy and *n* = 3 without therapy) and six *Dsg2^mt/wt^* mice (*n* = 3 with therapy and *n* = 3 without therapy).

Hearts were fixed after macroscopic inspection in 4% formaldehyde in phosphate buffered saline (PBS), dehydrated in a graded isopropanol series, and embedded in paraffin by routine procedures. The 5 µm paraffin sections were stained with hematoxylin/eosin and Heidenhain’s Azan trichrome stain to investigate cardiac morphology.

### 2.7. Immunohistochemical Analyses of the Extracellular Matrix Proteins Tenascin C and Transforming Growth Factor-Beta-Induced Protein Ig-h3 and the CD44 Antigen

To analyze subtle signs of cardiac remodeling/repair and inflammation, the localization of the matricellular protein tenascin C (TnC) and the CD44 protein, which is expressed on inflammatory cells, were studied in paraffin sections of a large proportion of the one-year-old mutant and wild-type mice (*n* = 6/13 *Dsg2^mt/wt^* and *n* = 3/9 *Dsg2^WT^* matched control mice; *n* = 9/7 *Dsg2^0/wt^* mice without structural phenotype, *n* = 3/2 *Dsg2^0/wt^* mice with structural phenotype and *n* = 11/6 *Dsg2^WT^* matched control mice). In addition, CD44 protein levels were analyzed in trained animals after Langendorff analysis (*n* = 4 for *Dsg2^WT^* with therapy and *n* = 3 for *Dsg2^WT^* without therapy, as well as *n* = 3 for *Dsg2^mt/wt^* mice with and without therapy).

After antigen retrieval (30 min in 10 mM citrate buffer, pH 6, 94 °C in a water bath), the sections were treated with blocking solution (ZytoChem-Plus-HRP-Polymer Kit, ZytomedSystems, Berlin, Germany) and incubated with the polyclonal rabbit TnC antibody diluted 1:2000 in PBS/10% fetal calf serum (FCS). The sections were covered with horse radish peroxidase (HRP)-coupled polymer according to the kit protocol (ready to use; ZytomedSystems, Berlin, Germany) and finally visualized with 3,3′-diaminobenzidine (DAB)/H_2_O_2_. The sections were counterstained with hematoxylin and mounted with glycerol gelatine.

The CD44 antigen was detected using a rat monoclonal antibody (Clone IM7, BD Pharmingen, San Diego, CA, USA; cat. No. 550538) diluted 1:50 in PBS (overnight incubation at 4 °C). Prior to the application of the CD44 antibody an antigen retrieval was performed (10 mM citrate buffer, pH 6, for 40 min at 94 °C in a water bath). The CD44 antibody was detected using the Histofine Simple Stain Mouse MAX PO (Rat) Kit (Medac, Wedel, Germany; cat. no. 414311F). Cells expressing CD44 were visualized with DAB/H_2_O_2_, and the sections were counterstained with hematoxylin and mounted with glycerol gelatin.

Cardiac transforming growth factor-beta-induced protein Ig-h3 (TGFBI) protein levels were studied in hearts obtained from animals after training and Langendorff analysis using a rabbit monoclonal antibody (Abcam, Cambridge, UK; ab169771). After antigen retrieval (10 mM citrate buffer, pH 6, incubation time 30 min at 94 °C in a water bath) and blocking of unspecific antibody binding with blocking solution (ZytoChem-Plus-HRP-Polymer Kit) the sections were incubated with the diluted antibody (1:250 in PBS) for one hour at room temperature. After washing, the specifically bound anti-TGFBI antibody was detected with an HRP-coupled polymer (ready to use; ZytoChem-Plus-HRP-Polymer Kit) and finally visualized with DAB/H_2_O_2_. The sections were counterstained with hematoxylin and mounted with glycerol gelatine.

The specificity of the first antibody and the detecting polymer system were controlled by replacing the first antibody with either non-immune serum or non-immune IgG of the same isotype, as well as by omitting the primary antibody. Cardiac sections of 4-week-old homozygous *Dsg2^mt/mt^* mice presenting nascent myocardial scars served as positive controls (Supplemental Figure S1A–C).

### 2.8. Connexin-43 Immunofluorescence Staining

Connexin-43 (CX43) protein distribution was assessed on 5 µm thick paraffin sections of one-year-old *Dsg2^0/wt^* (*n* = 12 with normal heart morphology and *n* = 3 with small fibrotic scars) and *Dsg2^mt/wt^* mice (*n* = 8) and their corresponding controls (*n* = 11 and *n* = 6, respectively). After dewaxing and rehydration, the sections were subjected to antigen retrieval (10 mM citrate buffer, pH 6; 3 min in a pressure cooker under full pressure). The anti-CX43 antibody (Sigma, St. Louis, MO, USA; cat. No. C6219) was diluted 1:1000 in PBS/1,5% bovine serum albumin (BSA) and applied overnight at 4 °C. After three washes with Tris wash buffer (300 mM NaCl, 50 mM Tris/HCl, pH 7.5, 0.045% (*v*/*v*) Tween-20), the secondary antibody (goat-anti rabbit conjugated with ALEXA 488 [Invitrogen, Waltham, MA, USA; A-11070]) was applied in a dilution of 1:500 in PBS/1.5% BSA for one hour at room temperature. After three washes in Tris wash buffer, the sections were incubated for 30 min in 0.1% Sudan Black in 70% ethanol to quench background fluorescence. After three washes in Tris wash buffer, the sections were counterstained with Hoechst 33,342 (2 µg/mL) and embedded with Mowiol 4-88 [42]. CX43 antibody specificity was tested by replacing the CX43 antibody with non-immune IgG and omission of the CX43 antibody (Supplemental Figure S1D,E). The score for the quantification of CX43 mislocalization was determined as follows (Table 1).

First, the number of cells with CX43 mislocalization was counted within the entire cardiac section (score N). Thereafter, the number of CX43-positive intracellular dots (score D) and the extent of lateral plasma membrane staining (score L) were determined. Finally, the mislocalization score S_CX43 mis_ was calculated using the following formula:

S_CX43 mis_ = score N × score D + score L

### 2.9. Transmission Electron Microscopy (TEM)

The cardiac ultrastructure was assessed by transmission electron microscopy (TEM) as described earlier [50]. In brief, hearts of *Dsg2^mt/wt^* and *Dsg2^WT^* mice aged 3 months (samples from both ventricles; *n* = 3 for each group), 5 months (right ventricular samples: *n* = 4 for each group), and 6–8 months (right ventricular samples: *n* = 3 for each group) were excised after the hearts were perfused via the left cardiac chamber with relaxation buffer (30 mM KCl, 5% glucose, 2 mL per animal) directly after cervical dislocation. Cardiac tissue samples were immediately minced into 1–2 mm^3^ pieces in fixative consisting of 4% formaldehyde + 1% glutaraldehyde in PBS. After 2 h fixation, the tissue samples were first treated for 1 h with 1% OsO_4_ and then for 2 h with 0.5% uranylacetate dissolved in 0.05 M sodium maleate buffer (pH 5.2) in the dark. After dehydration, the samples were incubated in acetone and embedded in araldite. Polymerization was carried out at 60 °C for 48 h. Ultrathin sections were prepared with a microtome (Ultracut S, Reichert, Depew, NY, USA). To enhance contrast, the sections were first treated with 3% uranyl acetate for 5 min and then with 0.08 M lead citrate solution for 4 min. Pictures were taken on an EM 10 (Zeiss, Oberkochen, Germany) with the MegaView III CCD camera (Olympus, Tokyo, Japan) using the iTEM software (ITEM_D_06March2007; Olympus, Tokyo, Japan).

### 2.10. Immunoblot Analysis of Cardiac Samples

The hearts of trained *Dsg2^mt/wt^* and *Dsg2^WT^* mice were harvested after Langendorff perfusion. LV and RV were separated and stored in liquid nitrogen for immunoblotting. LV and RV were pulverized in liquid nitrogen (BioPulverizer, Stratech Scientific, Cambridge, UK), mixed with warm 2 × SDS sample buffer (concentration 1×: 62.5 mM Tris/HCl pH 6.8, 100 mM DTT, 2% (*w*/*v*) SDS, 6% (*v*/*v*) glycerol), 0.02% (*w*/*v*) bromophenolblue; 4 µL per mg tissue), incubated at 65 °C, sonicated (BandelinSonoplus UW2070, Berlin, Germany) 5 s at 10% maximal output, and centrifuged at 17,000× *g* for 10 min at room temperature (RT). Supernatants (i.e., protein lysates) were diluted five-fold in 1.5 × SDS sample buffer, and loading was adjusted according to Coomassie-stained denaturing polyacrylamide gels. Proteins were resolved by SDS-PAGE (7.5% or 10% polyacrylamide) in a Mini-Protean 3 (Bio-Rad, Hercules, CA, USA) and transferred onto nitrocellulose (Protran BA83, GE Healthcare, Chicago, IL, USA) using a wet transfer system (Trans-Blot Cell, Bio-Rad, Hercules, CA, USA; transfer buffer 20% methanol in 1 × SDS running buffer containing 25 mM Tris base, 250 mM glycine, 0.1% (*v*/*v*) SDS, pH 8.8) at 250 mA and 4 °C for 16 h. Transfer and even loading were assessed by Ponceau S (Sigma, St. Louis, MO, USA) staining. The membranes were blocked with 5% non-fat dry milk (Marvel, New York, NY, USA) in TBST (150 mM NaCl, 50 mM Tris/HCl, pH 7.5, 0.1% (*v*/*v*) Tween-20) for 30 min at RT followed by primary antibody incubation overnight at 4 °C in blocking solution (Table 2). After washing with TBST (3 × 5 min), the membranes were incubated with species-specific secondary antibodies linked to horseradish peroxidase for 1 h at RT (anti-mouse HRP (cat # NA931VS) or anti-rabbit HRP (cat #NA934VS), both from GE Healthcare). The membranes were washed with TBST (containing 0.5% (*v*/*v*) Tween-20, 3 × 20 min) and incubated with SuperSignal West Pico or Dura Chemiluminescent Substrate (Thermo Scientific, Waltham, MA, USA). The films (Amersham Hyperfilm ECL, GE Healthcare, Chicago, IL, USA) were exposed and developed (Xograph Compact X4, Stonehouse, UK).

The DSG2 and plakophilin-2 levels were assessed on separate blots, whereas desmoplakin, beta-catenin, and plakoglobin were detected on one blot, which was cut into two pieces after Ponceau S staining. Cardiac actin was used as the internal control by using its characteristic band of 40 kDa on the Ponceau S-stained blots.

DSG2 protein levels of sedentary *Dsg2^0/wt^* and *Dsg2^WT^* mice were assessed as previously published [42]. In brief, hearts were Dounce-homogenized in extraction buffer (10 mM Tris-HCl pH 8.0, 2 mM MgCl_2_, 10 mM KCl, 2% SDS supplemented with a complete mini protease inhibitor tablet per 10 mL [Roche, Basel, Switzerland]). A quantity of 35 µg of protein was used per lane. After separation by SDS-PAGE, the proteins were transferred onto a PVDF membrane by tank blotting. The antibody dilutions were 1:2500 for polyclonal rabbit anti-DSG2 antibody (Rabbit-88) and polyclonal guinea pig anti-DSG2 (Gp 58) antibody. After overnight incubation at 4 °C and washing, the membranes were incubated with HRP-coupled anti-guinea pig antibodies diluted 1:5000 (Jackson Laboratory, Bar Harbor, ME, USA). β-actin served as the loading control. The bound antibodies were detected with an ECL prime kit (GE Healthcare–Chicago, IL, USA) and the Fusion SL imaging system (Vilber Lourmat, Eberhardzell, Germany).

### 2.11. Statistical Analyses

When CX43 mislocalization was compared between three groups (*Dsg2^WT^*, *Dsg2^0/wt^* without phenotype, and *Dsg2^0/wt^* with phenotype), the Kruskal–Wallis test and post hoc Dunn’s multiple comparison tests were applied. When CX43 mislocalization was analyzed between one-year-old *Dsg2^WT^* and *Dsg2^mt/wt^* mice, the Mann–Whitney test was utilized. The statistics were calculated with GraphPad Prism 9.

To compare the incidence of arrhythmias between *Dsg2^WT^* and *Dsg2^mt/wt^*, the Fishers exact test was used. When maximal activation time during LD between *Dsg2^WT^* and *Dsg2^mt/wt^* was compared, the unpaired *t*-test was used. Sidak’s multiple comparisons test was used to compare echocardiographic parameters between *Dsg2^WT^* with placebo and *Dsg2^mt/wt^* with placebo and *Dsg2^WT^* with preload-reducing therapy and *Dsg2^mt/wt^* with preload-reducing therapy before and after training. Tukey’s multiple comparisons test was used to compare the echocardiographic parameters of *Dsg2^WT^* with the placebo, *Dsg2^mt/wt^* with the placebo, and *Dsg2^WT^* with *Dsg2^mt/wt^* under preload-reducing therapy. The statistics were calculated with GraphPad Prism 8.3.

All experiments and primary analyses were blinded to the genotype, protocol step, and age group. Values were reported as mean ± standard error of the mean (SEM) unless indicated otherwise. Differences were considered significant (*) at a two-tailed alpha level of *p* < 0.05.

## 3. Results

### 3.1. Cardiac Morphology and Electrophysiology of Sedentary One-Year-Old Haploinsufficient Dsg2^0/wt^ and Heterozygous Dsg2^mt/wt^ Mutant Mice

At first, we studied whether sedentary *Dsg2^0/wt^* and *Dsg2^mt/wt^* mice developed an ARVC-like phenotype at the age of one year, which was comparable to a human age of 40–45 years [58,60].

#### 3.1.1. Hearts of One-Year-Old Sedentary Haploinsufficient *Dsg2^0/wt^* Mice

**Figure 2:**
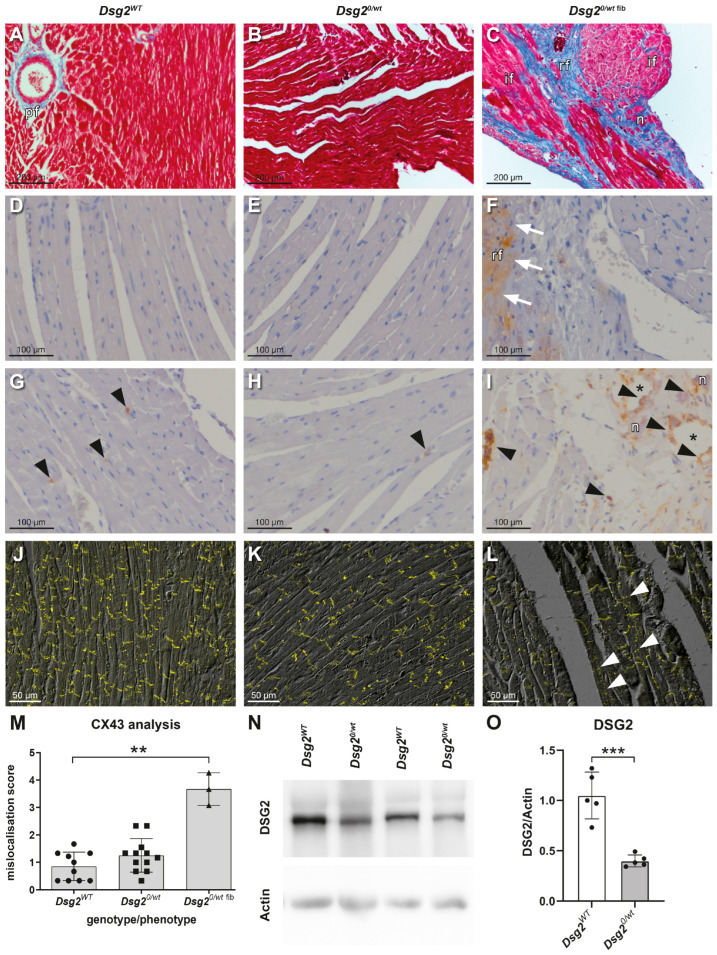
A structural ARVC phenotype is detectable only in 8% of one-year-old *Dsg2^0/wt^* haploinsufficient mice. (**A**,**D**,**G**,**J**) Hearts of one-year-old *Dsg2^WT^* mice, (**B**,**E**,**H**,**K**) heart sections of haploinsufficient *Dsg2^0/wt^* mice without a structural phenotype, and (**C**,**F**,**I**,**L**) heart sections of one-year-old *Dsg2^0/wt^* mice displaying an ARVC-like structural phenotype (*Dsg2^0/wt^* ^fib^ mice; 8% of mice with *Dsg2* haploinsufficiency). (**A**–**C**) Heidenhain’s AZAN staining detects fibrosis and scars (blue: pf = physiological periarterial fibrosis; if = interstitial fibrosis; rf = replacement fibrosis; n = necrotic cardiomyocytes). (**D**–**F**) Tenascin C immunostaining (arrows) highlights early cardiac remodeling. (**G**–**I**) CD44 immunostaining detects active inflammation (arrowheads indicate CD44 immune cells; asterisks (*) mark areas with necrotic and calcified cardiomyocytes, which appear empty due to material loss during heat-mediated antigen retrieval). (**J**–**M**) CX43 mislocalization to the lateral plasma membrane and CX43-positive cytoplasmic dots are detected predominantly in *Dsg2^0/wt^* mice with a structural phenotype (white arrowheads; ** *p* < 0.01). (**N**,**O**) Immunoblot analysis reveals reduced cardiac DSG2 protein levels in haploinsufficient *Dsg2^0/wt^* mice compared with mice harboring two functional *Dsg2* alleles (*** *p* < 0.001). Extended data are shown in Supplemental Figure S2, and original immunoblots are depicted in Supplemental Figure S3.

At the age of one year (52.6 ± 3.4 weeks), most *Dsg2^0/wt^* hearts did not show a significant chamber dilation or fibrotic wall changes upon autopsy. Neither Heidenhain’s AZAN trichrome nor the expression of Tenascin C (TnC), a sensitive marker for tissue repair in ARVC hearts [64], differed between the majority of wild-type and *Dsg2^0/wt^* hearts (Figure 2A,B,D,E and Supplemental Figure S2). But 8% of *Dsg2^0/wt^* hearts showed a mild dilation of the RV and small white ventricular lesions. At the histological level these lesions presented as replacement fibrosis from which strands of interstitial fibrosis emanated (*Dsg2^0/wt^* ^fib^ in Figure 2C). Ongoing fibrotic remodeling was indicated by TnC expression (Figure 2F).

To search for signs of inflammation, CD44 immunostaining was performed. Hearts of one-year-old *Dsg2^0/wt^* and wild-type mice showed single CD44-positive cells either scattered throughout the myocardium or in the direct vicinity of arteries and the epicardium (Figure 2G,H and Supplemental Figure S2). In *Dsg2^0/wt^* hearts with ventricular lesions, CD44-positive cells accumulated adjacent to and within the fibrotic scar tissue (Figure 2I), whereas in the unaffected myocardium only a few scattered CD44-positive cells were present.

CX43 immunofluorescence staining did not differ between one-year-old *Dsg2^0/wt^* hearts without fibrosis and *Dsg2^WT^* hearts (Figure 2J,K,M). However, significant CX43 lateralization and an increased number of CX43-positive cytoplasmic dots were detected in cardiomyocytes adjacent to fibrotic lesions of *Dsg2^0/wt^* ^fib^ hearts (Figure 2L,M) as we have previously observed in cardiac-specific *Dsg2* knockout mice [42]. DSG2 protein levels were significantly reduced in *Dsg2^0/wt^* hearts compared with those of hearts expressing the wild-type DSG2 as shown by immunoblot analysis (Figure 2N,O).

The ECG and echocardiographic parameters of the studied sedentary one-year-old *Dsg2^0/wt^* and *Dsg2^WT^* mice did not differ significantly (Table 3 and Table 4).

#### 3.1.2. Hearts of One-Year-Old Sedentary *Dsg2^mt/wt^* Mice

**Figure 3:**
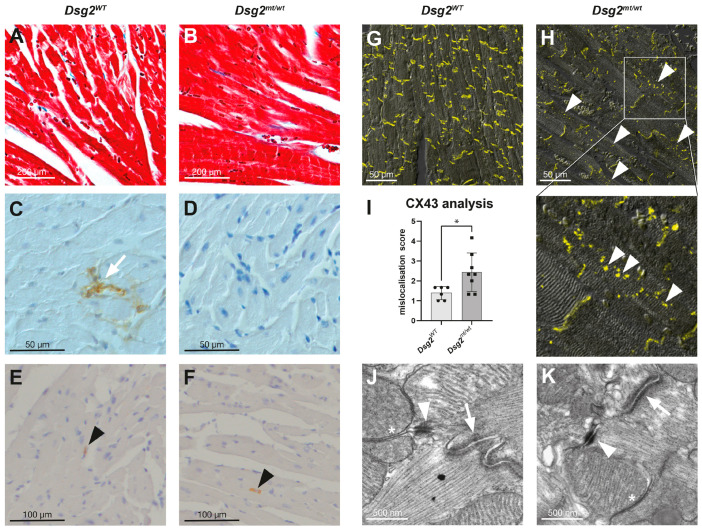
Sedentary heterozygous one-year-old *Dsg2^mt/wt^* mice have a higher incidence of CX43 mislocalization than their wild-type counterparts but do not show cardiac fibrosis or inflammation. (**A**,**B**) Representative Heidenhain’s Azan trichrome-stained sections of one-year-old *Dsg2^mt/wt^* (**A**) and *Dsg2^WT^* (**B**) hearts. The results of connective tissue semiquantification are presented in Supplemental Figure S2. (**C**,**D**) Only single foci of TnC-positive interstitial cells (arrow) were detected within entire heart cross sections of *Dsg2^WT^* and *Dsg2^mt/wt^* hearts. (**E**,**F**) CD44-positive immune cells (arrowheads) were rarely found in the hearts of one-year-old *Dsg2^WT^* and *Dsg2^mt/wt^* mice. (**G**–**I**) CX43 immunolocalization. *Dsg2^mt/wt^* hearts harbor more cardiomyocytes with mislocalized CX43 (arrowheads; (**H**) with image detail magnified 2.6 fold) than wild-type hearts (* *p* < 0.05). (**J**,**K**) Transmission electron microscopy reveals that desmosomes (white triangles) are discernable in *Dsg2^mt/wt^* hearts. White arrows, fascia adherens; *, gap junctions. Extended data are shown in Supplemental Figure S5.

We previously studied *Dsg2^mt/wt^* mice up to an age of three months. During this period of life, they did not present myocardial fibrosis, inflammatory infiltrates, or gene expression indicative of cardiac remodeling or failure [48]. In the present study we now found that the cardiac morphology of sedentary one-year-old *Dsg2^mt/wt^* mice (53.1 ± 6.2 weeks) did not differ noticeably from that of the wild-type littermates. Furthermore, Heidenhain’s AZAN staining did not reveal differences in the extent of connective tissue between both genotypes (Figure 3A,B; for semiquantitative assessment, refer to Supplemental Figure S4). Even when using TnC expression as a sensitive marker for myocardial remodeling, no obvious differences were detected between *Dsg2^mt/wt^* and *Dsg2^WT^* hearts (Figure 3C,D and Supplemental Figure S5). Furthermore, CD44 immunohistochemistry did not reveal enhanced inflammatory activity either in *Dsg2^mt/wt^* or in *Dsg2^WT^* hearts (Figure 3E,F). However, CX43 mislocalization was detected in significantly more cardiomyocytes of *Dsg2^mt/wt^* than *Dsg2^WT^* hearts (Figure 3G–I).

Desmosome morphology was then analyzed in five- to eight-month-old *Dsg2^mt/wt^* and *Dsg2^WT^* mice (Figure 3J,K). Desmosomal structure seemed to be preserved in *Dsg2^mt/wt^* hearts.

### 3.2. Hearts of 16- to 29-Week-Old Sedentary Dsg2^mt/wt^ Mice

Next, we assessed heart morphology and function in resting 16- and 26-week-old *Dsg2^mt/wt^* and *Dsg2^WT^* mice using echocardiography (Table 5). No significant differences were detected between the genotypes. In addition, neither ECG recordings nor Langendorff examinations of isolated hearts indicated genotype-related deviations (Table 6, left columns), indicating that the *Dsg2* mutation was fully compensated in sedentary mice.

### 3.3. Induction of an ARVC-like Phenotype in Heterozygous Dsg2^mt/wt^ Mice by Endurance Swim Training

To test whether endurance swim training provoked an ARVC phenotype in heterozygous *Dsg2^mt/wt^* mutant mice, young adult *Dsg2^mt/wt^* and wild-type control mice were subjected to seven weeks of incremental swim training ([61], Supplemental Table S1).

#### 3.3.1. Electrical Phenotype in *Dsg2^mt/wt^* Mice after Endurance Swim Training

ECGs were recorded after endurance swim training. The duration of the S_min_-J_peak_ interval was significantly increased in the trained *Dsg2^mt/wt^* mice receiving the placebo compared with the sedentary group and with the trained *Dsg2^mt/wt^* mice receiving the preload therapy (Table 6, Supplemental Figure S6).

**Figure 4:**
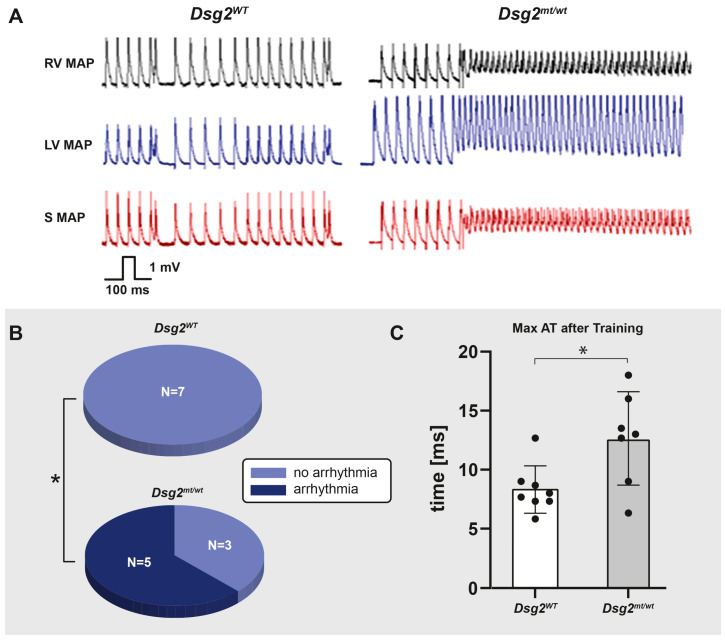
Incremental endurance swim training increases the inducibility of ventricular arrhythmias and prolongs the maximal activation time in *Dsg2^mt/wt^* hearts. (**A**) Representative examples of original monophasic action potential (MAP) recordings from *Dsg2^WT^* (**left**) and *Dsg2^mt/wt^* (**right**) Langendorff-perfused hearts after endurance swim training. Ventricular tachycardia (VT) is induced by a single right ventricular extra stimulus S2 during right ventricular pacing in a *Dsg2^mt/wt^* heart (RV MAP (black): MAP measured in the RV wall; LV MAP (blue): MAP measured in the left ventricular wall; S MAP (red): MAP measured in the septum). (**B**) Number of Langendorff-perfused hearts with arrhythmias (dark blue) and without arrhythmias (light blue) in trained *Dsg2^WT^* and *Dsg2^mt/wt^* mice induced by a single right ventricular extra stimulus during right ventricular pacing show increased arrhythmia events in *Dsg2^mt/wt^* mice (*Dsg2^WT^*: *n* = 0 of *n* = 7; *Dsg2^mt/wt^*: *n* = 5 of *n* = 8; * *p* < 0.05). (**C**) Maximal ventricular activation time (max AT) measured in Langendorff-perfused *Dsg2^WT^* (blank) and *Dsg2^mt/wt^* (gray) hearts with a pacing cycle length of 100 ms. The activation time was prolonged in *Dsg2^mt/wt^* hearts compared with *Dsg2^WT^* hearts (*n* = 8 for *Dsg2^WT^*, *n* = 7 for *Dsg2^mt/wt^*; * *p* < 0.05).

Furthermore, hearts from *Dsg2^mt/wt^* mice showed increased susceptibility to ventricular arrhythmias induced by a single extra stimulus during assessments on the Langendorff apparatus after swim training (5/8 trained *Dsg2^mt/wt^* mice, 0/7 trained *Dsg2^WT^* mice; *p* < 0.05; Figure 4A,B). Ventricular activation times during right ventricular pacing were increased in the trained *Dsg2^mt/wt^* hearts (Figure 4C).

#### 3.3.2. Structural and Functional Phenotype in *Dsg2^mt/wt^* Mice after Endurance Swim Training

**Figure 5:**
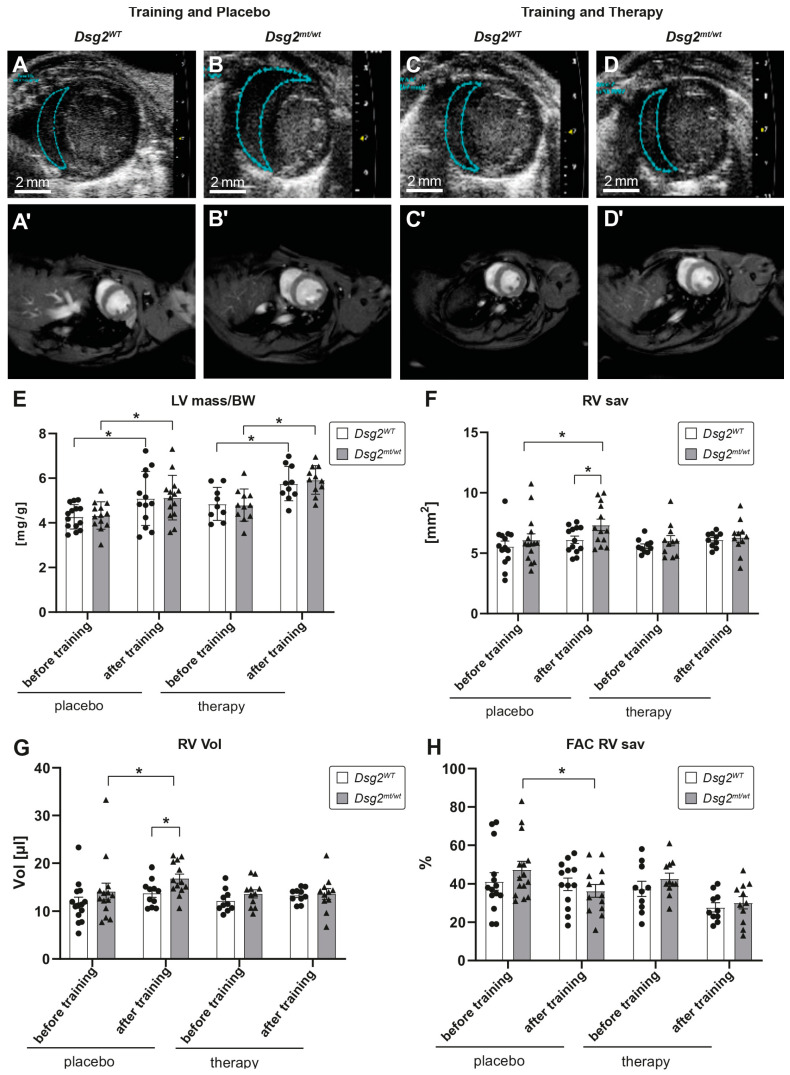
Right ventricular enlargement in *Dsg2^mt/wt^* mice after endurance swim training is alleviated by preload-reducing therapy. (**A**–**D**) Representative echocardiographic images of the short-axis view during diastole after endurance swim training of *Dsg2^WT^* (**A**) and *Dsg2^mt/wt^* (**B**) mice treated with placebo and *Dsg2^WT^* (**C**) and *Dsg2^mt/wt^* (**D**) mice with preload-reducing treatment therapy. The blue dotted line surrounds the area measured of the right ventricle in the short-axis view. (**A’**–**D’**) MRI showing prospectively triggered cardiac Cine magnetic resonance images acquired at 9.4 T (groups and protocols as in (**A**–**D**)). (**E**) Bar graphs showing increased left ventricular mass/body weight (LVmass/BW) index after endurance swim training without (placebo) and with preload-reducing therapy (* *p* < 0.05). Augmentation of the index provides evidence that the training was effective. (**F**) Bar graphs representing the measured diastolic right ventricular (RV) area in the short axis view during echocardiography before and after training, without (placebo) and with preload reduction therapy in *Dsg2^WT^* and *Dsg2^mt/wt^* mice (* *p* < 0.05). After training, *Dsg2^mt/wt^* mice showed an increased RV short axis area. (**G**) Bar graphs showing calculated diastolic RV volume before and after training, without (placebo) and with preload reduction therapy in *Dsg2^WT^* and *Dsg2^mt/wt^* mice (* *p* < 0.05). After training, RV volume was enlarged in trained and placebo-treated *Dsg2^mt/wt^* mice compared with before training and with trained and untreated *Dsg2^WT^* mice. (**H**) Contractility of the RV shown by the fractional area change (FAC) measured in the short axis view (sav) before and after training, with placebo and with therapy in *Dsg2^WT^* and *Dsg2^mt/wt^* mice (* *p* < 0.05). After training, FAC RV sav was reduced in trained *Dsg2^mt/wt^* mice.

Swim training induced mild left ventricular wall hypertrophy and an increased left ventricular mass/body weight index in the *Dsg2^mt/wt^* and *Dsg2^WT^* mice (Figure 5E), in line with the mild QRS prolongation with training (Table 6).

Echocardiography revealed enhanced right ventricular end diastolic dimensions and an increased right ventricular end diastolic volume (Figure 5F,G) in the trained *Dsg2^mt/wt^* mice compared with the trained *Dsg2^WT^* mice. The right ventricular dimensions were also increased compared with the pre-training measurements. Swim training reduced right ventricular function in *Dsg2^mt/wt^* mice, calculated by the right ventricular fractional area change during diastole and systole (RV FAC sav; Figure 5H), but not in wild-type controls. Neither left ventricular diameters nor functions differed between *Dsg2^mt/wt^* and *Dsg2^WT^* mice after training (Table 7).

### 3.4. Preload Reduction Therapy Diminished Development of RV Dilation in Dsg2^mt/wt^ Mice

We have previously shown that preload-reducing therapy by nitrates and diuretics [61] decreased the RV enlargement induced by endurance swim training in *Dsg2^mt/wt^* mice (Figure 5F,G). Preload-reducing therapy did not prevent training-induced LV hypertrophy (Figure 5E). The LV function was not altered between genotypes and compared with the untreated group (Table 7).

### 3.5. Cardiac Histology of Dsg2^WT^ and Dsg2^mt/wt^ Mice after Endurance Swim Training

**Figure 6:**
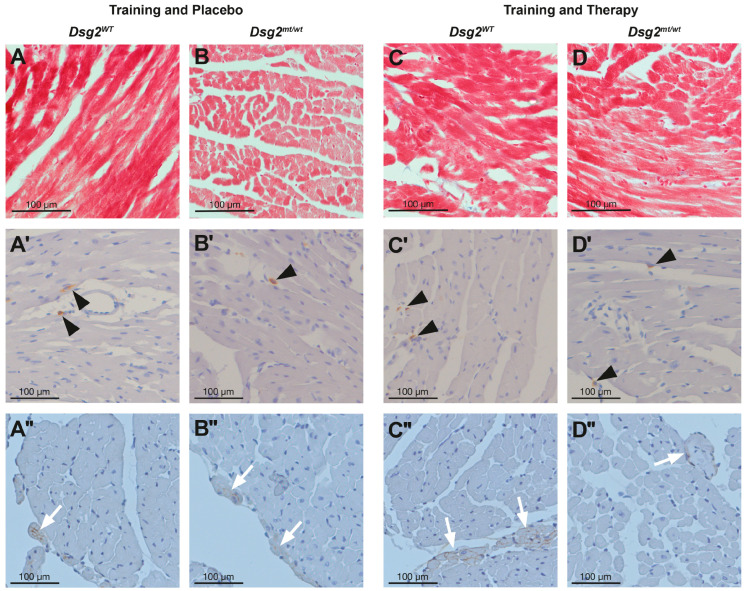
Hearts of trained *Dsg2^mt/wt^* mice show no obvious signs of fibrosis and/or inflammation in immunohistochemistry. (**A**–**D**) Representative images of cardiac sections stained with Heidenhain’s AZAN staining. (**A’**–**D’**) The typical localization of the sporadically detected CD44-positive inflammatory cells (black triangles). (**A”**–**D”**) Occasionally found foci of TGFBI protein expression (white arrows). The foci are typically located near or within the sites of heart valve attachment, whereas the endomysium and perimysium of the myocardium are devoid of TGFBI staining indicating the absence of fibrotic myocardial remodeling in these regions. Extended data are shown in Supplemental Figure S7.

Cardiac histology was studied in the *Dsg2^mt/wt^* and *Dsg2^WT^* mice after the endurance swim training with and without the preload-reducing therapy after completion of Langendorff assessments. There were no detectable fibrotic changes after seven weeks of endurance swim training (Figure 6A’–D’) and no overt activation of fibrotic remodeling as verified by immunostaining of the matricellular protein TGFBI, which was upregulated during healing after cardiac infarction (Figure 6A”–D”, Supplemental Figure S7) [65]. Furthermore, we did not detect an influx of CD44-positive inflammatory cells in the myocardium of the *Dsg2^mt/wt^* mice with and without therapy (Figure 6A”–D”, Supplemental Figure S7). These analyses suggest that the training-induced right ventricular dilation in the *Dsg2^mt/wt^* mice (Figure 5) may be related to the direct cellular effects of the mutant DSG2 protein.

### 3.6. Desmosomal Protein Contents

**Figure 7:**
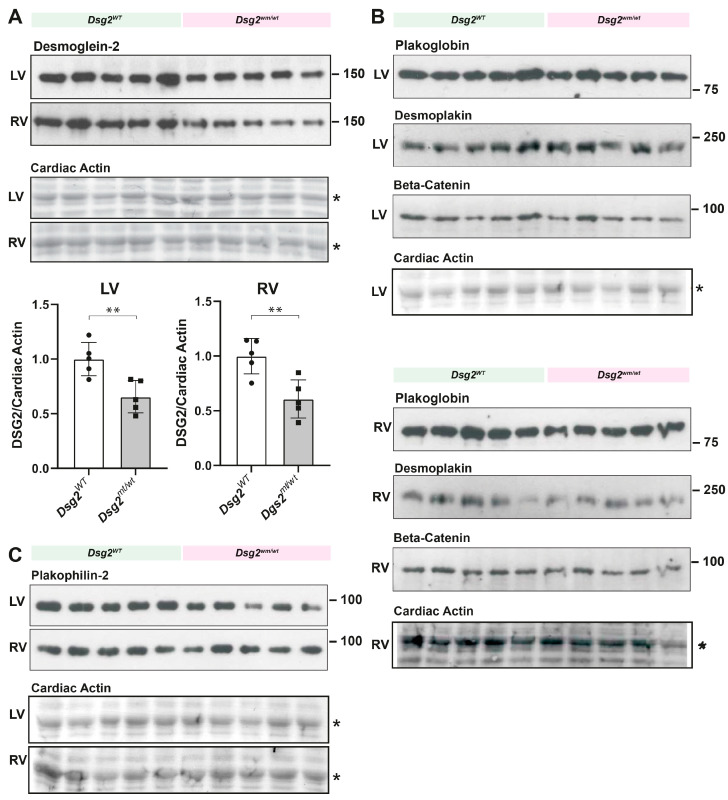
DSG2 protein content is reduced in *Dsg2^mt/wt^* hearts after training, whereas the amount of the desmosomal proteins plakoglobin, plakophilin-2, and desmoplakin and the fascia adherens protein beta-catenin is not altered. (**A**) Immunoblots showing DSG2 and, as a control, cardiac actin levels (* on Ponceau S stain) in the left ventricle (LV) and right ventricle (RV) of *Dsg2^WT^* and *Dsg2^mt/wt^* hearts after endurance swim training. Bar graphs show the relative amounts of DSG2 normalized to cardiac actin. In the LV as well as in the RV, *Dsg2^mt/wt^* hearts showed a lower DSG2 level. Bar graphs present the mean ± SEM. *n* = 5 per genotype and heart chamber; ** *p* < 0.007. (**B**) Immunoblots showing the amount of the desmosomal proteins plakoglobin, desmoplakin, and beta-catenin in LVs and RVs of *Dsg2^WT^* and *Dsg2^mt/wt^* hearts after training. (**C**) Immunoblot showing plakophilin-2 protein levels in left and RVs of *Dsg2^WT^* and *Dsg2^mt/wt^* hearts after training. The amount of these proteins is unaffected in *Dsg2^mt/wt^* hearts compared with wild-type hearts (*n* = 5 per genotype and heart chamber). The original immunoblots are shown in Supplemental Figure S8.

As shown for the resting *Dsg2^0/wt^* hearts (Figure 2N,O), the DSG2 protein content was reduced in *Dsg2^mt/wt^* hearts after training (Figure 7A), whereas the amount of other desmosomal proteins like plakoglobin, plakophilin-2, and desmoplakin and the fascia adherens protein beta-catenin were not altered (Figure 7B,C). Although the DSG2 protein was reduced in both ventricles, training-induced ventricular dilation occurred only in the RV.

## 4. Discussion

This study analyzed the effects of aging, endurance swim training, and preload-reducing therapy in conjunction with endurance swim training in heterozygous *Dsg2^mt/wt^* mice and gained four new findings:

i. Aging with a sedentary lifestyle does not provoke RV dilation or ECG alterations but may still cause CX43 mislocalization in heterozygous *Dsg2^mt/wt^* mice.

ii. Heterozygous *Dsg2* mutation leads to a training-induced ARVC-like phenotype with RV enlargement and RV arrhythmias in young adult mice.

iii. Preload-reducing therapy alleviates the training-induced ARVC phenotype.

iv. The training-induced ARVC phenotype of *Dsg2^mt/wt^* mice does not entail detectable fibrosis or inflammatory infiltrate.

These results are similar to our findings in plakoglobin haploinsufficient mice [61], emphasizing that a preload-reducing therapy is a feasible therapeutic approach in ARVC based on different desmosomal defects.

### 4.1. Endurance Swim Training Induces ARVC-like Right Ventricular Pathologies in Dsg2^mt/wt^ Hearts

The seven-week-long incremental endurance swim training provoked a dilation of the RV and a susceptibility to arrhythmia in the trained *Dsg2^mt/wt^* mice, whereas the wild-type mice remained unaffected. Endurance training results in an elevated blood flow, increased right ventricular preload and afterload, and elevated RV end systolic wall stress [29], whereas the LV wall is much less affected. Even in healthy athletes without known genetic variants, prolonged extreme endurance exercise may lead to fatigue and structural remodeling of the RV [66]. Our data show that the training-induced cardiac workload exceeds the compensatory capacity of the RV in mice harboring a dysfunctional *Dsg2* allele and thus results in an early ARVC-like phenotype. Our data are in line with studies showing that endurance training induces an early ARVC phenotype in other mouse models heterozygous for a mutant or haploinsufficient for a desmosomal protein [33,61,67]. They are furthermore in line with the observations in human ARVC patients harboring one mutant desmosomal gene [28,29,30,31,32]. However, we observed neither striking fibrotic changes nor an elevated number of CD44-positive inflammatory cells in trained *Dsg2^mt/wt^* mice. The lack of pronounced histopathology was also described by us for the haploinsufficient plakoglobin mouse [33,61] and for wild-type mice with AAV-driven cardiac expression of mutant plakophilin-2 [68]. The lack of histological alterations makes them good models for the early concealed phase of ARVC.

### 4.2. Preload-Reducing Therapy Prevents Right Ventricular Dilation

ARVC is known to be a progressive disease [4]. Exercise restriction has been shown to slow the progression and is one component of clinical ARVC management recommended in international guidelines. To date, therapeutic approaches are symptomatic and comprise cardioverter defibrillator implantation, catheter ablation, and/or beta-blocker and antiarrhythmic agents [34,69]. As mechanical overload and wall stress of the right ventricular walls are known as a progressive mechanism in ARVC patients, a preload-reducing therapy could be a promising therapeutic tool. Our data together with our previous observations [61] suggest that preload reduction during a period of endurance swim training prevents right ventricular enlargement in mutant *Dsg2* allele carriers.

### 4.3. The Effect of Aging on Disease Development in Dsg2^0/wt^ and Dsg2^mt/wt^ Mice

In addition to exercise, aging is an established second hit that promotes ARVC manifestation not only in humans but also in mice carrying mutant desmosomal genes [61,68,70]. We therefore studied whether *Dsg2* haploinsufficient or heterozygous mutant *Dsg2* mice develop an overt ARVC phenotype during aging. The age effect was examined in mice aged one year, which was equivalent to a human age of 40–50 years [58,60]. Patients with a mild ARVC disease course are commonly diagnosed at this age [31,71]. While the great majority of the one-year-old *Dsg2^0/wt^* and *Dsg2^mt/wt^* mice did not develop the typical histomorphological ARVC phenotype detected in heart-specific *Dsg2* knockout or homozygous *Dsg2^MT^* mice [42,48,50], the genetic predisposition for ARVC was revealed by some one-year-old *Dsg2^0/wt^* mice with small fibrotic lesions accompanied by an inflammatory infiltrate and CX43 mislocation in lesion-near cardiomyocytes. Carriers of desmosomal gene mutations are at risk to be affected by life-threatening arrhythmias even before morphological alterations are established [31,72]. However, neither the ECG nor the electrophysiological parameters were affected significantly in morphologically inconspicuous *Dsg2^0/wt^* mice, implying that one functional *Dsg2* gene may be sufficient to support normal cardiac function in a sedentary lifestyle, at least up to the age of one year in mice. In contrast, we reported that 10-month-old sedentary haploinsufficient plakoglobin mice showed ventricular arrhythmias, although they were structurally inconspicuous [33]. The pathophysiology behind these differences is partially explained by the fact that individual desmosomal proteins are connected to different cellular signaling pathways [20,21,73].

### 4.4. Limitations

All our findings are limited to murine models, which, however, enabled the observation of training and treatment-induced effects in vivo under controlled conditions. Validation in patients is still needed. Our findings report the effects of endurance swim training and of preload reduction in a second murine model of ARVC. These findings support the clinical evaluation of preload-reducing therapy in patients with ARVC. While our histological techniques have been successfully applied to detect fibrotic remodeling and influx of inflammatory cells in the heart, our experiments cannot fully rule out small changes in inflammatory or profibrotic regulation. Due to the limited number of *Dsg2^0/wt^* mice and the methods applied, we may have missed subtle early electrical and morphological changes.

### 4.5. Conclusions

Endurance swim training induces an ARVC-like phenotype in otherwise healthy and morphologically inconspicuous *Dsg2^mt/wt^* mice presenting with RV dilation, decreased RV contractility, and increased inducibility of ventricular arrhythmias during right ventricular pacing. Prolonged ventricular activation times present a possible electrical mechanism for increased arrhythmia risk. RV dilation was prevented by preload-reducing therapy. Heterozygous *Dsg2*-mutant mice serve as useful models for the assessment of the concealed phase of ARVC.

### 4.6. Clinical Perspective

While exercise restriction is recommended for patients, when ARVC is fully diagnosed according to the task force criteria, there is still a debate about what guidance should be given to gene carriers and relatives with the same pathogenic variant as a patient with a full disease phenotype. Observations in murine models with a mutant *Dsg2* gene suggest that long-duration swim training can provoke an ARVC phenotype when there is a genetic risk for a desmosomal disease. When preload reduction was shown to prevent ARVC in plakoglobin haploinsufficient mice, the questions arose whether this effect could be confirmed for other murine ARVC models. Here we show that preload reduction prevents ARVC also in mice with a heterozygous *Dsg2* defect. In addition, a retrospective safety study was able to show the safety of preload reduction in patients with ARVC [74]. This calls for controlled trials of preload reduction in ARVC patients alone or in combination with novel interventions targeting genes and desmosomal structures directly.

## Figures and Tables

**Table 1:** Histological CX43 score.

Number of Cells with Mislocalized CX43		Number of CX43^+^ Intracellular Dots		Lateral Plasma Membrane Staining	
Cell Number	Score N	Dot Number per Cell	Score D	Extent	Score L
0	0	0	0	0	0
1–5	1	1–5	1	1–10%	1
6–10	2	6–10	2	11–20%	2
11–20	3	>11	3	≥30%	3
>20	4				

**Table 2:** Primary and secondary antibodies used on immunoblots.

Protein	Source	Clone/Cat. No.	Species	Dilution	ECL Kit
Plakoglobin	BD Biosciences, Franklin Lakes, NJ, USA	15/γ-Catenin	Mouse	1:25,000	Dura
Plakoglobin	Sigma, St. Louis, MO, USA	15F11	Mouse	1:500	Dura
Plakophilin-2	Progen, Heidelberg, Germany	PP2/62, 2/86, 2/150	Mouse	1:300	Dura
Desmoplakin	Serotec, Kidlington, UK	AHP320	Rabbit	1:2000	Dura
β-catenin	Sigma, St. Louis, MO, USA	C2206	Rabbit	1:700	Pico
DSG2	[63]	Rb-88	Rabbit	1:2000	Dura
DSG2	Generated by Peptide Specialty Laboratories, Heidelberg, Germany	Gp 57, Gp 58	Guinea pig	1:2500	ECL prime
α-actinin	P. van der Ven	653	Rabbit	1:5000	Pico
β-actin	Sigma, St. Louis, MO, USA	A2066	Rabbit	1:2000	ECL prime

**Table 3:** ECG parameters of one-year-old, sedentary *Dsg2^0/wt^* and *Dsg2^WT^* mice.

Genotype	*Dsg2^WT^*	*Dsg2^0/wt^*
**N**	4	5
**Age (weeks)**	53 ± 0	53 ± 0
**HR (bpm)**	601 ± 27	619 ± 14
**PQ interval (ms)**	38 ± 1	39 ± 1
**P-wave duration (ms)**	16 ± 1	16 ± 1
**QRS duration (ms)**	17 ± 1	17 ± 1
**QT interval (ms)**	53 ± 3	52 ± 2

**Table 4:** Echocardiographic parameters of one-year-old, sedentary *Dsg2^0/wt^* and *Dsg2^WT^* mice.

Genotype	*Dsg2^WT^*	*Dsg2^0/wt^*
**N**	6	6
**Age (weeks)**	51 ± 0.6	51 ± 0.5
**BW (g)**	35 ± 1	34 ± 2
**Heart rate (bpm)**	446 ± 11	490 ± 17
**RVsav (mm^2^)**	5.98 ± 0.7	6.46 ± 0.9
**RV4 (mm^2^)**	5.7 ± 1.0	5.9 ± 0.6
**RVDV (µL)**	15.4 ± 2.6	15.7 ± 2.4
**RV FAC sav (%)**	36.7 ± 2	41.1 ± 4
**LVEDV (µL)**	81.7 ± 5.6	96.8 ± 8.4
**IVSd (mm)**	0.8 ± 0.0	0.8 ± 0.0
**LVPWd (mm)**	0.9 ± 0.0	0.8 ± 0.0
**LV FAC sav (%)**	53.6 ± 2	46.3 ± 4
**LV Mass/BW (mg/g)**	4.1 ± 0.3	4.4 ± 0.4
**LVEDV/RVDV (µL/µL)**	5.7 ± 1.7	6.9 ± 1.1

Values are mean ± SEM. BW = bodyweight; RV = right ventricle; RVsav = right ventricle area short axis view; RV4 = right ventricle area four-chamber view; RVDV = right ventricle diastolic volume (calculated by Wübbelling formula); RV FAC sav = calculation of right ventricular fractional area change (areas measured in short axis view); LV = left ventricle; LVEDV = left ventricle volume; IVSd = thickness of diastolic interventricular septal wall; LVPWd = thickness of diastolic left ventricular wall; LV FAC sav = calculation of left ventricular fractional area change (areas measured in short axis view); LV mass = calculated mass of left ventricle.

**Table 5:** Echocardiographic parameters of sedentary, 16- and 26-week-old *Dsg2^mt/wt^* and *Dsg2^W^*^T^ mice.

	16 Weeks		26 Weeks	
Sedentary	*Dsg2^WT^*	*Dsg2^mt/wt^*	*Dsg2^WT^*	*Dsg2^mt/wt^*
**N**	9	11	11	11
**BW (g)**	27.9 ± 1.7	25.1 ± 1.3	25.5 ± 1.3	27.9 ± 0.7
**Heart rate (bpm)**	492 ± 197	492 ± 8.5	439 ± 13.4	429 ± 10.4
**RVsav (mm^2^)**	5.0 ± 0.3	5.5 ± 0.4	6.8 ± 0.4	6.9 ± 0.4
**RV4 (mm^2^)**	7.6 ± 0.3	8.8 ± 0.5	7.6 ± 0.3	8.5 ± 0.4
**RVDV (µL)**	9.7 ± 0.7	11.8 ± 1.1	13.8 ± 1.2	14.5 ± 1.2
**RV FAC sav (%)**	35.6 ± 6	32.9 ± 3	37.2 ± 5	39.1 ± 2
**LVEDV (µL)**	71.6 ± 3.0	65.5 ± 2.7	81.1 ± 4.0	88.4 ± 6.7
**IVSd (mm)**	0.9 ± 0.0	0.9 ± 0.0	0.6 ± 0.0	0.7 ± 0.1
**LVPWd (mm)**	0.9 ± 0.0	0.8 ± 0.0	0.7 ± 0.0	0.7 ± 0.0
**LV FAC sav (%)**	43.9 ± 1	47.0 ± 2	47.3 ± 2	49.7 ± 3
**LV Mass/BW (mg/g)**	4.8 ± 0.2	4.8 ± 0.1	4.0 ± 0.4	4.0 ± 0.3
**LVEDV/RVDV (µL/µL)**	7.4 ± 0.4	6.0 ± 0.5	6.1 ± 0.4	6.2 ± 0.3

**Table 6:** ECG data of *Dsg2^WT^* and *Dsg2^mt/wt^* mice aged 29 weeks, which served as sedentary control group, and trained *Dsg2^mt/wt^* and *Dsg2^WT^* mice with placebo or preload-reducing therapy.

Protocol	Sedentary		Training			
			Placebo		Preload-Reducing Therapy	
Genotype	*Dsg2^WT^*	*Dsg2^mt/wt^*	*Dsg2^WT^*	*Dsg2^mt/wt^*	*Dsg2^WT^*	*Dsg2^mt/wt^*
**N**	7	8	12	13	5	10
**Age (weeks)**	29 ± 0.4	29 ± 0.4	21 ± 0.9	20 ± 0.8	18 ± 0.4	19 ± 0.3
**HR (bpm)**	554 ± 23	569 ± 36	528 ± 20	579 ± 20	523 ± 23	603 ± 30
**PQ interval (ms)**	38.1 ± 1	36.4 ± 0	36.1 ± 1	35.7 ± 1	34.6 ± 1	**33.8 ± 1 #**
**P wave duration (ms)**	15.1 ± 1	16.5 ± 0	16.3 ± 1	17.1 ± 0 ^+^	14.7 ± 1	15.7 ± 0
**QRS duration (ms)**	13.3 ± 0	13.9 ± 0	**14.5 ± 0 #**	14.7 ± 0	13.7 ± 0	13.8 ± 0
**S_min_-J_peak_ interval (ms)**	4.5 ± 0	4.5 ± 0	4.9 ± 0	**5.7 ± 0 #^+^**	4.4 ± 0	4.6 ± 0
**QT interval (ms)**	48.4 ± 1	47.6 ± 4	46.3 ± 2	49.6 ± 2	49.8 ± 4	45.1 ± 2

# *p* < 0.05 no training vs. training, same genotype; + *p* < 0.05 placebo vs. treated, same genotype.

**Table 7:** Echocardiographic parameters of *Dsg2^mt/wt^* and *Dsg2^WT^* mice before and after swim training without (placebo) and with preload-reducing therapy.

	Placebo				Preload-Reducing Therapy			
	Before Training		After Training		Before Training		After Training	
	*Dsg2^WT^*	*Dsg2^mt/wt^*	*Dsg2^WT^*	*Dsg2^mt/wt^*	*Dsg2^WT^*	*Dsg2^mt/wt^*	*Dsg2^WT^*	*Dsg2^mt/wt^*
**N**	14	15	14	14	10	11	10	11
**Heart rate** (bpm)	449 ± 10.5	449 ± 8.3	419 ± 9.9	433 ± 9.1	465 ± 7.4	457 ± 9.72	424 ± 8.5 *	437 ± 9.6
**RVsav** (mm^2^)	5.6 ± 0.4	6.1 ± 0.5	6.1 ± 0.3	7.3 ± 0.5 #*	5.6 ± 0.2	6.0 ± 0.4	6.1 ± 0.2	6.3 ± 0.42
**RV4** (mm^2^)	7.0 ± 0.3	7.5 ± 0.4	8.3 ± 0.6	8.2 ± 0.6	7.9 ± 0.3	8.3 ± 0.3	9.6 ± 0.3 *	9.2 ± 0.5
**RVDV** (µL)	11.8 ± 1.2	14.1 ± 1.7	13.7 ± 0.8	16.8 ± 1.0 #*	12.2 ± 0.8	13.6 ± 0.8	13.3 ± 0.5	13.6 ± 1.1
**RV FAC sav** (%)	41.3 ± 5	47.4 ± 4	39.8 ± 3	36.4 ± 3 *	37.3 ± 4	42.9 ± 3	27.8 ± 2	30.2 ± 3
**RV EF (%)** **^§^**	51 ± 2 8 (*n* = 5)	52 ± 3 (*n* = 4)	51 ± 3 (*n* = 10)	50 ± 3 (*n* = 10)	60 ± 3 (*n* = 10)	54 ± 3 (*n* = 9)	54 ± 4 (*n* = 10)	54 ± 3 (*n* = 10)
**LVEDV** (µL)	70.1 ± 3.1	76.9 ± 3.9	70.5 ± 3.6	71.8 ± 3.5	73.7 ± 4.4	72.0 ± 3.1	69.5 ± 3.8	65.1 ± 2.8
**IVSd** (mm)	0.7 ± 0.0	0.7 ± 0.0	0.9 ± 0.1 *	0.9 ± 0.0 *	0.7 ± 0.0	0.8 ± 0.0	1.1 ± 0.1 *+	1.1 ± 0.0 *+
**LVPWd** (mm)	0.7 ± 0.0	0.7 ± 0.0	0.8 ± 0.1	0.8 ± 0.0 *	0.7 ± 0.0	0.7 ± 0.0	0.7 ± 0.0	0.8 ± 0.0 *
**LV FAC sav** (%)	56.1 ± 2	55.8 ± 4	49.5 ± 4	47.1 ± 3	50.9 ± 3	54.5 ± 2	47.3 ± 3	50.9 ± 2
**LV EF** (%)	54 ± 2	51 ± 2	60 ± 4	57 ± 3	48 ± 1	53 ±2	52 ± 2	55 ± 2
**LV mass/BW** (mg/g)	4.3 ± 0.2	4.3 ± 0.2	5.1 ± 0.3 *	5.2 ± 0.3 *	4.9 ± 0.3	4.8 ± 0.2	5.8 ± 0.2 *	5.9 ± 0.9 *
**LVEDV/RVDV** (µL/µL)	6.5 ± 0.5	6.2 ± 0.4	5.2 ± 0.4	4.4 ± 0.2 *	6.4 ± 0.29	5.4 ± 0.3	5.2 ± 0.2 *	5.0 ± 0.47

Values are mean ± SEM; * *p* < 0.05 before versus after training, # *p* < 0.05 *Dsg2^WT^* versus *Dsg2^wt/mt^*, + *p* < 0.05 untreated versus treated same genotype, ^§^ RV EF% was not determined in all mice of the studied cohort; RV = right ventricle; RVsav = right ventricle area short axis view; RV4 = right ventricle area four-chamber view; RVDV = right ventricle diastolic volume (calculated by Wübbelling formula); RV FAC sav = calculation of right ventricular fractional area change (areas measured in short axis view); RV EF = calculation of right ventricular ejection fraction (volumes calculated from diastolic and systolic four-chamber view measurements); LV = left ventricle; LVEDV = left ventricle volume; IVSd = thickness of diastolic interventricular septal wall; LVPWd = thickness of diastolic left ventricular wall; LV FAC sav = calculation of left ventricular fractional area change (areas measured in short axis view); LV EF = calculation of the left ventricular ejection fraction (calculated from volumes measured in the long axis view); LV mass = calculated mass of left ventricle.

## Data Availability

More detailed data are available in the Supplement. Further supporting reported results will be made available upon reasonable request.

## Supplementary Materials

The following supporting information can be downloaded at https://www.mdpi.com/article/10.3390/biomedicines12050985/s1, Figure S1: Results of positive and negative control experiments of immunostainings; Figure S2: Results of semiquantitative assessment of interstitial and periarterial cardiac fibrosis; Figure S3: Original immunoblots to determine cardiac DSG2 protein levels in haploinsufficient *Dsg2* mice; Figure S4: Semiquantitative assessment of interstitial and periarterial cardiac fibrosis in 12-week-old and one-year-old *Dsg2^mt/wt^* and *Dsg2^WT^* mice; Figure S5: Right ventricular distribution of single CD44-positive cells and rare TGFBI-positive areas (supplement to Figure 3); Figure S6: Prolonged right ventricular activation is indicated by late ventricular activation in untreated *Dsg2^mt/wt^* mice after training (see Table 6); Figure S7: The micrographs show the right ventricular distribution of CD44-positive cells and TGFBI-positive areas (supplement to Figure 5); Figure S8: Original immunoblots (supplement to Figure 7); Table S1: Time schedule of swim training.
