# Supplementary material for: Endurance Training Provokes Arrhythmogenic Right Ventricular Cardiomyopathy Phenotype in Heterozygous Desmoglein-2 Mutants: Alleviation by Preload Reduction
Source: Biomedicines. 2024 Apr 30;12(5):985. doi: 10.3390/biomedicines12050985 (PMC11117820; doi:10.3390/biomedicines12050985)
Supplement: Supplementary file 1 [file biomedicines-12-00985-s001.zip › Supplemental Figures and Tables -biomedicines-2714893.pdf]

Supplemental Figures and Tables Fabritz et al., "Endurance Training Provokes Arrhythmogenic Right Ventricular Cardiomyopathy Phenotype in Heterozygous Desmoglein-2 Mutants: Alleviation by Preload Reduction"

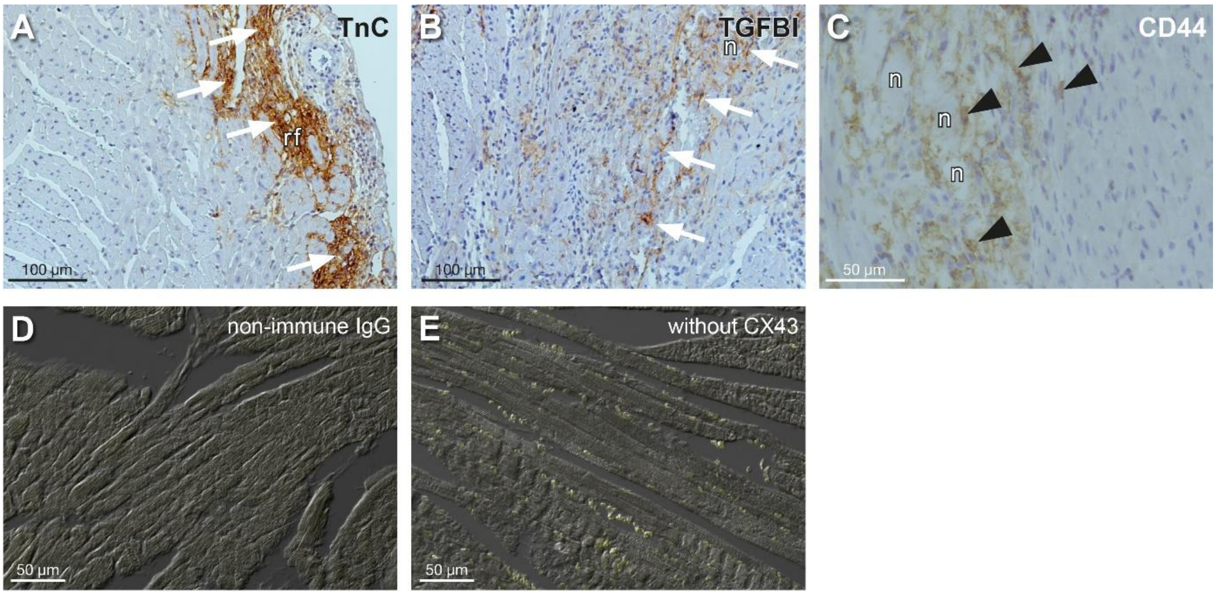

Supplemental Figure S1 depicts the results of representative positive and negative control immunostainings of Tenascin C (TnC), TGFBI and CX43.
(**A**) white arrows point to TnC positive replacement fibrosis (rf) in the heart of a homozygous *Dsg2* mutant mouse and in (**B**) white arrows point to TGFBI positive connective tissue surrounding necrotic (n) cardiomyocytes of a homozygous *Dsg2* mutant mouse. (**C**) black arrowheads indicate CD44 positive cells in direct neighborhood of necrotic cardiomyocytes (n) surrounded by connective tissue in a homozygous *Dsg2* mutant mouse. (**D, E**) depict negative control reactions for the CX43 immunofluorescence staining: (**D**) first antibody was replaced by non-immune IgG and (**E**) the first antibody was omitted.

Supplemental Table S1 summarizes the schedule of group swim training and the time points of cardiac function analyses.

	start	Incremental swim training 5 - 90 min swimming / d: [experimental weeks]							end
experimental schedule [weeks]	Echo	1	2	3	4	5	6	7	end of 7th week Echo and ECG Langendorff analyses
Dsg2$^{mt/wt}$	N=11	with preload reducing therapy							N=11
Dsg2$^{mt/wt}$	N=14	without therapy (placebo)							N=14
Dsg2^{WT}	N=10	with preload reducing therapy							N=10
Dsg2^{WT}	N=14	without therapy (placebo)							N=14

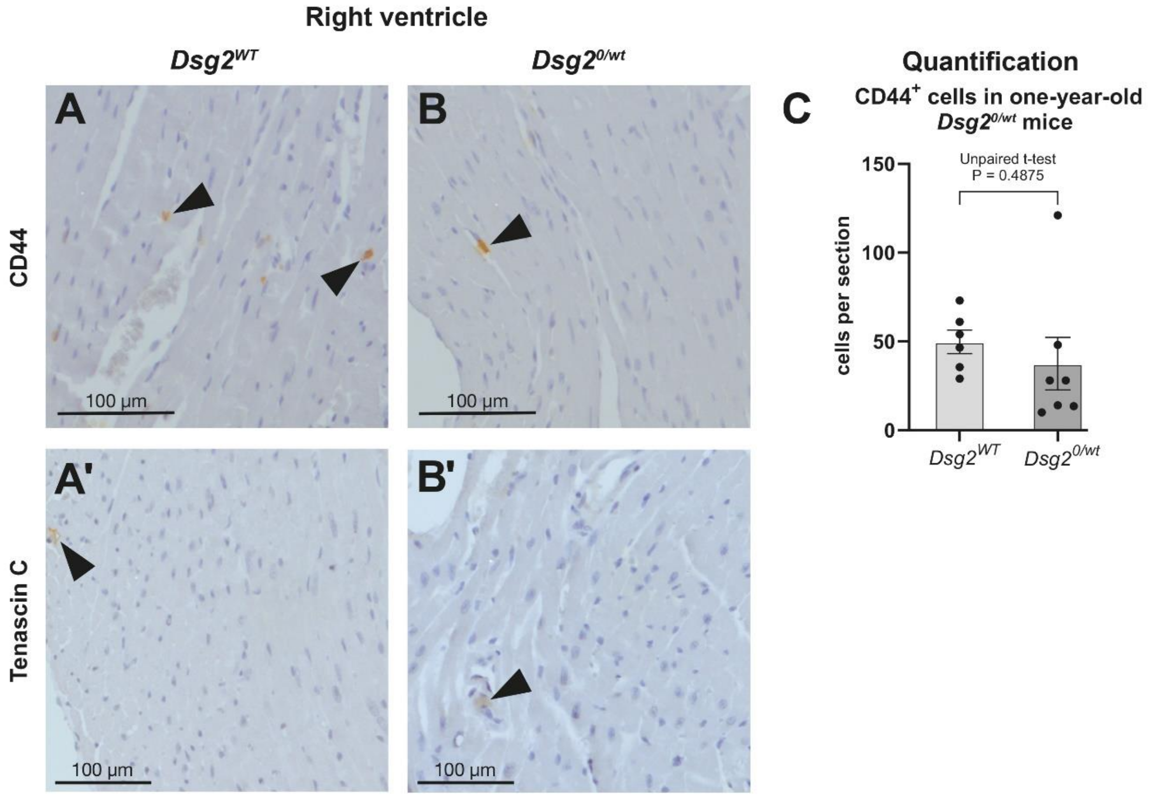

Supplemental Figure S2 (to Figure 2) shows the right ventricular distribution of single CD44 positive cells and the rare Tenascin C positive areas in *Dsg2$^{0/wt}$* and *Dsg2^{WT}* mice.
(**A, B**) Single scattered CD44 positive cells (arrowheads) are present in right ventricular walls of one-year-old *Dsg2^{WT}* (**A**) and *Dsg2$^{0/wt}$* mice (**B**). Rarely a cluster of 3-4 CD44 positive cells is found in periarterial connective tissue in both genotypes (not shown). (**C**) The amount of CD44 positive cells per cardiac cross section did not differ between genotypes.
(**A`, B`**) Tenascin C immunoreactivity was rarely detected and was restricted to tiny endomysial areas (arrowhead, **A`**) and/or periarterial areas (arrowhead, **B`**) in *Dsg2$^{0/wt}$* and *Dsg2^{WT}* mice. Limited Tenascin C immunoreactivity was also found at insertion sites of the cardiac valves in both genotypes (not shown) and rated as physiological staining. For pathological staining refer to Supplemental Figure S1A.

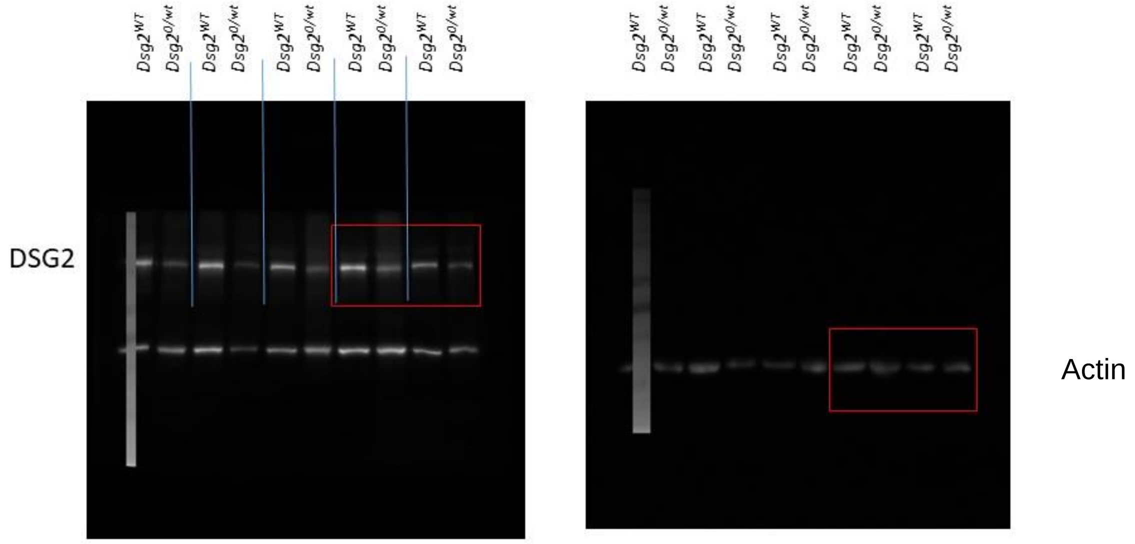

Supplemental Figure S3 (to Figure 2) shows the whole original immunoblots used to determine cardiac DSG2 protein expression in *Dsg2* wildtype (*$Dsg2^{WT}$*) and *Dsg2* haploinsufficient (*$Dsg2^{0/wt}$*) mice. The representative data presented in Figure 2 are marked by the red rectangles. The lower band in the DSG2 immunoblot is an unspecific band.

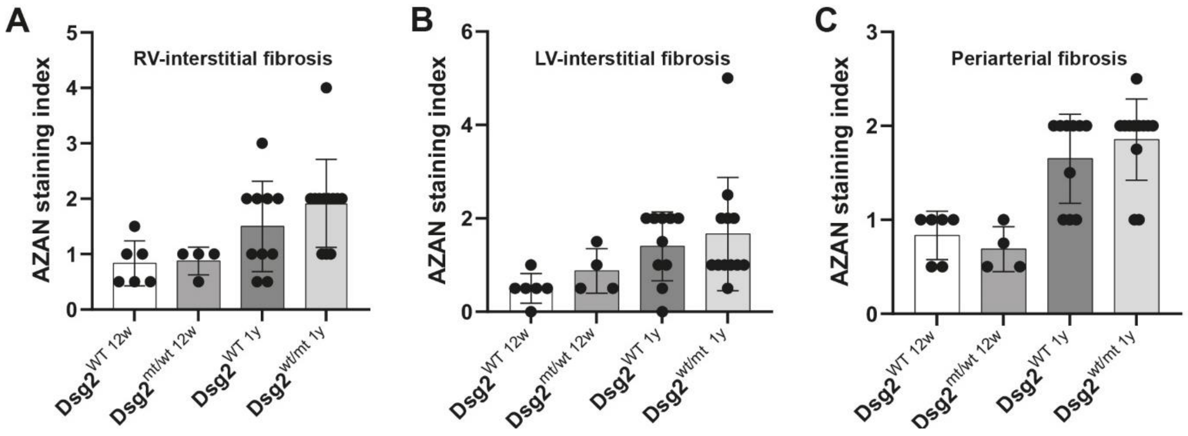

Supplemental Figure S4 presents the results of the semiquantitative assessment of interstitial and periarterial cardiac fibrosis in 12-week-old and one-year-old *Dsg2*$^{mt/wt}$ and *Dsg2*^{WT} mice. The fibrotic index was determined in serial cardiac sections of 12-week-old (*Dsg2*^{WT} $n = 6$ and *Dsg2*$^{mt/wt}$ $n = 4$) and one-year-old (*Dsg2*^{WT} $n = 10$ and *Dsg2*$^{mt/wt}$ $n = 12$) mice stained with Heidenhain`s Azan trichrome stain. At first sections were assessed using the 5× objective. Areas containing interstitial fibrosis were assessed by using the 20× objective. Values of 0 to 3 were given according to the extent of extracellular matrix accumulation with 0.5 increments. To rule out that perivascular fibrosis was misinterpreted as interstitial fibrosis serial sections were studied. Periarterial connective tissue was classified by the thickness and the density of the extracellular matrix surrounding the arteries. Periarterial fibrosis was scored with values from 0.5 to 3 in 0.5 increments.

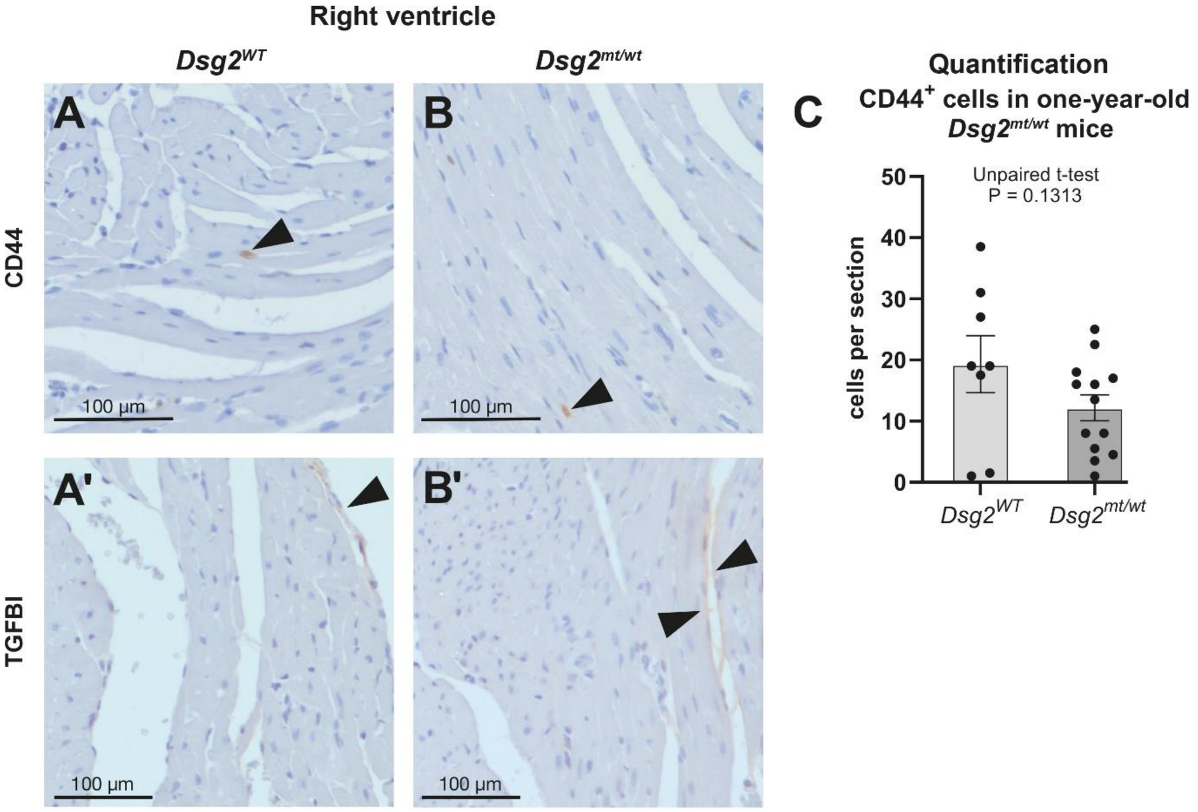

Supplemental Figure S5 (to Figure 3) shows the right ventricular distribution of single CD44 positive cells and the rare TGFBI positive areas.

(**A, B**) Single scattered CD44 positive cells (arrowheads) are present in right ventricular walls of one-year-old *Dsg2*^{WT} (**A**) and *Dsg2*$^{mt/wt}$ mice (**B**). Rarely a cluster of 3-4 CD44 positive cells is found in periarterial connective tissue in both genotypes (not shown). (**C**) The number of CD44 positive cells in cardiac cross sections did not differ between genotypes.

(**A`, B`**) TGFBI immunoreactivity was rarely detected (arrowheads) and was restricted to some regions of the epicardium (**A`**) and/or small, narrowly circumscribed areas of the perimysium (**B`**) in *Dsg2*$^{mt/wt}$ and *Dsg2*^{WT} mice. Little TGFBI immunoreactivity was also present in parts of the endocardium and at insertion sites of the cardiac valves in both genotypes (refer to Supplemental Figure S7) and rated as physiological staining. For pathological staining refer to Supplemental Figure S1B.

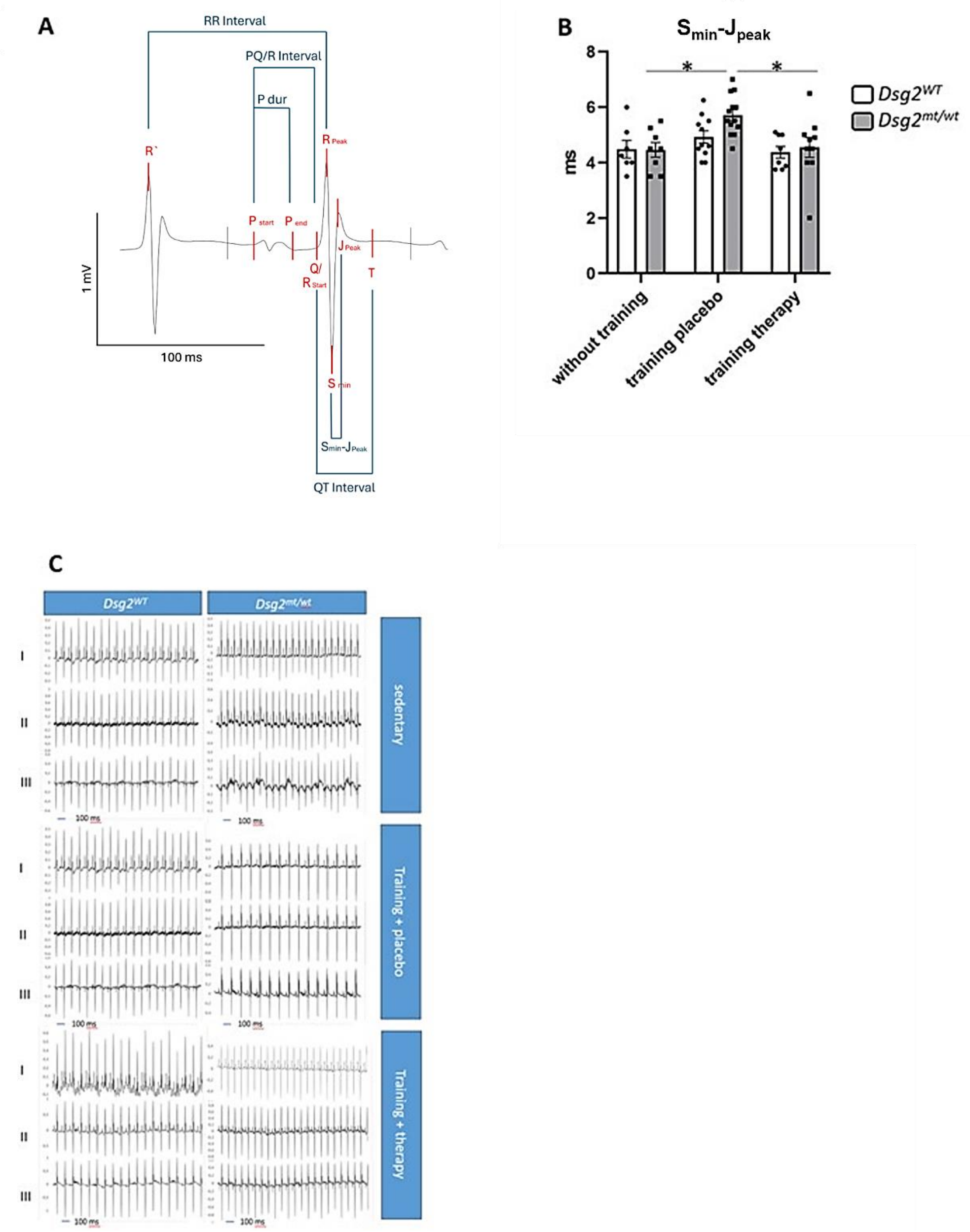

Supplemental Figure S6 (to Table 6) Prolonged right ventricular activation is reflected by late ventricular activation in untreated *Dsg2*$^{mt/wt}$ mice after training. (**A**) shows an example of a signal averaged ECG beat of lead III using 100 single beats of a surface ECG. Measuring points and measured intervals (values see Table 6) are indicated. (**B**) depicts a bar graph presenting late ventricular activation times (S_{min}-J_{peak}) that were determined in signal averaged ECGs recorded in untrained, trained placebo-treated and trained preload reducing therapy-treated *Dsg2*$^{mt/wt}$ (grey) and *Dsg2*^{WT} (white) mice. S_{min}-J_{peak} is prolonged in trained placebo-treated *Dsg2*$^{mt/wt}$ mice compared to untrained *Dsg2*$^{mt/wt}$ mice and trained *Dsg2*$^{mt/wt}$ mice that were subjected to preload reducing therapy (*p <0.05). (**C**) Representative examples of surface ECG leads I, II and III recorded in sedentary, trained placebo-treated and trained preload reducing therapy-treated (therapy) *Dsg2*$^{mt/wt}$ and *Dsg2*^{WT} mice.

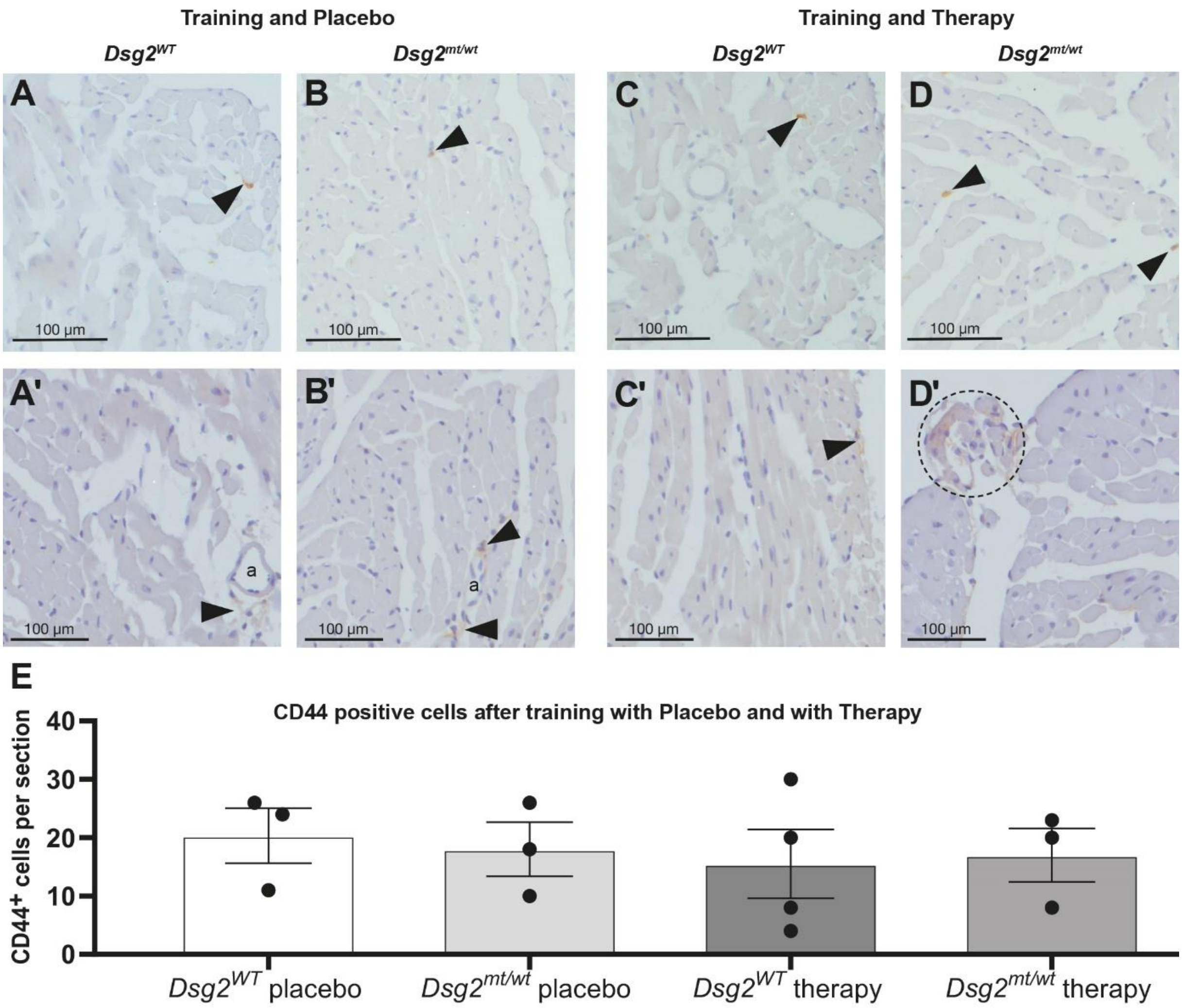

Supplemental Figure S7 (to Figure 6) shows the right ventricular distribution of CD44 positive cells and TGFBI positive areas in mice after endurance swim training with and without preload reduction therapy.
(**A-D**) Single scattered CD44 positive cells (arrowheads) are present in the right ventricular walls of trained *Dsg2^{WT}* and *Dsg2$^{mt/wt}$* mice either treated with placebo or with preload reducing therapy. Rarely small clusters of 3-4 CD44 positive cells were detected in periarterial connective tissue independent of genotype and treatment (not shown). (**E**) The number of CD44 positive cells per cardiac cross section did not differ between genotypes and treatments.
(**A`-D`**) TGFBI immunoreactivity was restricted to periarterial connective tissue (arrowheads in **A`** and **B`**; a = arteria), some random areas of the epicardium (arrowhead in **C`**) and insertion sites of cardiac valves (encircled in **D`**). No obvious differences were detected between genotypes and treatments.

Desmoglein-2 immunoblot

A blots 262 (left ventricle) and 263 (right ventricle) were incubated with the anti DSG2 antibody exposition time 15 min

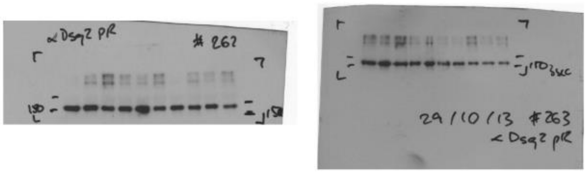

B Ponceau S stained blots Nr. 262 and 263

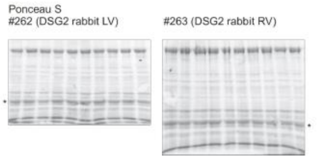

* Cardiac actin band

Beta-Catenin, Plakoglobin and Desmoplakin immunoblot

A blots 248 (left ventricle) and 249 (right ventricle) were cut and probed with beta catenin (βcat) exposition time 10 sec; anti-PG antibody exposition time 10 sec; anti DSP antibody exposition time o/n

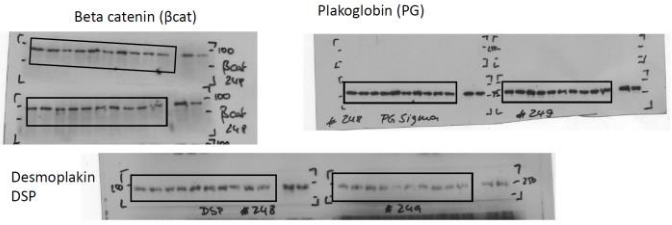

B Ponceau S stained blots Nr. 248 (left ventricle) and 249 (right ventricle) which were probed with beta-catenin plakoglobin and desmoplakin antibodies

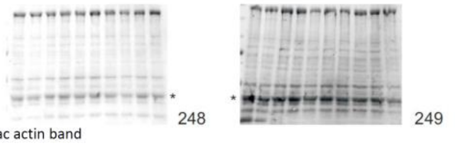

* Cardiac actin band

Plakophilin-2 immunoblot

A Blots 246 (left ventricle) and 247 (right ventricle) were cut and probed the anti PKP2 antibody, exposition time 1min

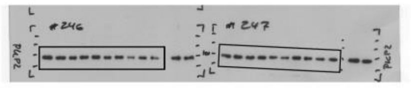

B Ponceau S stained blots Nr. 246 and 247 probed with PKP2

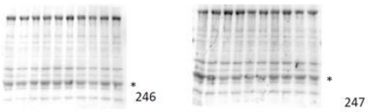

* Cardiac actin band

Supplemental Figure S8 (to Figure 7) shows the original immunoblots and the Ponceau S stained membranes.
